# Gasdermin B over-expression modulates HER2-targeted therapy resistance by inducing protective autophagy through Rab7 activation

**DOI:** 10.1186/s13046-022-02497-w

**Published:** 2022-09-26

**Authors:** Manuel Gámez-Chiachio, Ángela Molina-Crespo, Carmen Ramos-Nebot, Jeannette Martinez-Val, Lidia Martinez, Katja Gassner, Francisco J. Llobet, Mario Soriano, Alberto Hernandez, Marco Cordani, Cristina Bernadó-Morales, Eva Diaz, Alejandro Rojo-Sebastian, Juan Carlos Triviño, Laura Sanchez, Ruth Rodríguez-Barrueco, Joaquín Arribas, David Llobet-Navás, David Sarrió, Gema Moreno-Bueno

**Affiliations:** 1 Departamento de Bioquímica, Universidad Autónoma de Madrid (UAM) Instituto de Investigaciones Biomédicas ‘Alberto Sols’ (CSIC-UAM), IdiPAZ, C/Arturo Duperier 4, 28029 Madrid, Spain; 2 Centro de Investigación Biomédica en Red de Cáncer (CIBERONC), Instituto de Salud Carlos III, Madrid, Spain; 3 Departamento de Zoología Genética Antropología Física, Universidad Santiago de Compostela, Lugo, Spain; 4 Mecanismos Moleculares Y Terapia Experimental en Oncologia-Programa OncobellIdibell, L’Hospitalet de Llobregat, Spain; 5 Servicio de Microscopía Electrónica, Centro de Investigación Príncipe Felipe (CIPF), Valencia, Spain; 6 Servicio de Microscopía Óptica Avanzada, Centro de Investigación Príncipe Felipe (CIPF), Valencia, Spain; 7 Programa de Investigación Preclínica, Vall d’Hebron Institute of Oncology (VHIO), Barcelona, Spain; 8 Leitat Medical Department, Leitat Technological Center, Barcelona, Spain; 9 Fundación MD Anderson Internacional, Madrid, Spain; 10 Sistemas Genómicos, Paterna, Valencia Spain; 11 Departamento de Patologia Y Terapèutica Experimental Facultad de Medicina, Unidad de Anatomia Universidad de Barcelona (UB), L’Hospitalet de Llobregat, Spain; 12 Programa de Investigación en Cáncer, IMIM (Hospital del Mar Medical Research Institute), Barcelona, Spain; 13 Institució Catalana de Recerca I Estudis Avançats (ICREA), Barcelona, Spain

**Keywords:** Gasdermin B, Protective autophagy, Anti-HER2 therapy, Drug resistance, HER2 breast cancer, Gastroesophageal tumors, LC3B

## Abstract

**Background:**

Gasdermin B (GSDMB) over-expression promotes poor prognosis and aggressive behavior in HER2 breast cancer by increasing resistance to therapy. Decoding the molecular mechanism of GSDMB-mediated drug resistance is crucial to identify novel effective targeted treatments for HER2/GSDMB aggressive tumors.

**Methods:**

Different *in vitro* approaches (immunoblot, qRT-PCR, flow cytometry, proteomic analysis, immunoprecipitation, and confocal/electron microscopy) were performed in HER2 breast and gastroesophageal carcinoma cell models. Results were then validated using *in vivo* preclinical animal models and analyzing human breast and gastric cancer samples.

**Results:**

GSDMB up-regulation renders HER2 cancer cells more resistant to anti-HER2 agents by promoting protective autophagy. Accordingly, the combination of lapatinib with the autophagy inhibitor chloroquine increases the therapeutic response of GSDMB-positive cancers *in vitro* and in zebrafish and mice tumor xenograft *in vivo* models. Mechanistically, GSDMB N-terminal domain interacts with the key components of the autophagy machinery LC3B and Rab7, facilitating the Rab7 activation during pro-survival autophagy in response to anti-HER2 therapies. Finally, we validated these results in clinical samples where GSDMB/Rab7/LC3B co-expression associates significantly with relapse in HER2 breast and gastric cancers.

**Conclusion:**

Our findings uncover for the first time a functional link between GSDMB over-expression and protective autophagy in response to HER2-targeted therapies. GSDMB behaves like an autophagy adaptor and plays a pivotal role in modulating autophagosome maturation through Rab7 activation. Finally, our results provide a new and accessible therapeutic approach for HER2/GSDMB + cancers with adverse clinical outcome.

**Supplementary Information:**

The online version contains supplementary material available at 10.1186/s13046-022-02497-w.

## Background

Gasdermin B (GSDMB) is one of the six Gasdermin (GSDM) genes in the human genome (along with GSDMA, C, D, GSDME/DFNA5, and DFNB59/PJVK) [1]. Recent data suggest that all GSDMs, under specific circumstances, can trigger a lytic and pro-inflammatory cell death mechanism, known as pyroptosis [2, 3]. This pro-cell death function is usually auto-inhibited through the intramolecular interaction of GSDM N-terminal (NT) and C-terminal (CT) domains, but upon cleavage by specific caspases and other proteases, the released N-terminal domain forms membrane pores that subsequently lead to cell lysis [2, 3]. In the case of GSDMB, the pro-cell death function under physiological conditions is a matter of intense debate [4–7]. Recently, it has been revealed that lymphocyte-derived Granzyme-A can cleave GSDMB within tumor cells, thus provoking a pyroptotic cancer cell death [8]. In this sense, an activated anti-tumor immune response mediated by NK and T-cytotoxic cells could reduce tumor growth of murine colon and melanoma cells exogenously overexpressing GSDMB [8]. Moreover, GSDMB pore-forming activity can also have microbiocidal function during enterobacteria infection, pointing out a new role of GSDMB in host immunity [9]. However, GSDMB plays other diverse non-pyroptotic functions in inflammatory pathologies [10, 11] and cancer. In tumors, GSDMB is frequently over-expressed in breast, liver, colon, cervical and gastric carcinomas, and GSDMB upregulation can promote multiple pro-tumor functions [3].

In particular, GSDMB is frequently (> 60%) over-expressed in HER2 breast cancer, mostly due to GSDMB-HER2 co-amplification [12]. In these tumors, GSDMB over-expression and/or amplification, associates significantly with poor prognosis and reduced response to anti-HER2 standard therapy (trastuzumab) independently of the hormone receptor status or histological grade [12]. Moreover, GSDMB overexpression promotes cell motility, invasion, and metastasis in breast cancer cell lines [13, 14], but it does not affect cell proliferation [13]. Interestingly, our recent data showed that GSDMB over-expression is a novel therapeutic target in HER2 breast tumors [14] since the intracellular delivery of a GSDMB antibody using nanoparticles significantly reduces the tumor growth and metastasis development in HER2 breast tumors by inducing cancer cell death *in vivo* [14]. Given that HER2 amplification/overexpression also occurs frequently in gastroesophageal cancers [15], and considering that some cancer patients can experience drug resistance to diverse available anti-HER2-targeted therapies (antibodies like trastuzumab, pertuzumab, T-DM1; tyrosine kinase inhibitors (TKI) such as lapatinib neratinib or tucatinib) [16], we proposed that identifying the functional mechanisms by which GSDMB over-expression modulates the therapeutic response of different HER2 tumors could have novel clinical utility. Indeed, there is a growing necessity to discover the resistance mechanisms of the FDA-approved anti-HER2 TKIs and therapeutic strategies to overcome it, since they have advantageous characteristics (such as oral administration, capability to reach CNS metastasis, multiple HER family targets, lower cardiotoxicity), and importantly they are the therapeutic alternatives to the treatment of anti-HER2 antibodies-refractory HER2 + breast cancer patients [17]. Furthermore, diverse evidence showed that there is some cross-resistance to these three TKIs, thus being lapatinib resistant cells less sensitive to the others and the other way round [17–19].

To this end, here we demonstrate that GSDMB over-expression mediates pro-survival autophagy after anti-HER2 TKI treatment. While protective autophagy has been linked to cancer resistance to diverse HER2-targeted drugs [20] and autophagy inhibition can partially abrogate this effect [21, 22], the precise molecular mechanisms involved in this process are still unclear. Our data prove that the combination of anti-HER2 TKI treatment with the autophagy inhibitor chloroquine (CQ) increases the therapeutic response specially in GSDMB-positive tumors *in vitro* and *in vivo*. In addition, we show that GSDMB increases pro-survival autophagy through a cooperation of its N-terminal with the microtubule‑associated protein light chain 3B (LC3B), an essential modulator of the autophagic machinery [23, 24] and Rab7, a small GTPase involved in the autophagosome maturation [25, 26]. Overall, we have uncovered a novel functional link between GSDMB over-expression, Rab7 and LC3B, where GSDMB acts like an autophagy adaptor by inducing Rab7 activation and subsequently enhancing protective autophagy in response to HER2-targeted therapies. Besides, this work provides a new therapeutic approach for HER2/GSDMB + cancers, characterized by poor clinical outcome.

## Methods

### Human tumor samples

The present study was carried out using the 31 available HER2 breast carcinoma series treated with adjuvant regimen that was previously reported [12] and a novel intestinal gastric tumor cohort (*n* = 59). Gastric tumors were acquired from the Biobank of the Anatomy Pathology Department (record number B.0000745, ISCIII National Biobank network) of the MD Anderson Cancer Center Madrid, Madrid, Spain. The mean patient age at diagnosis was 59.8 ± 12.7 years (range, 29 to 85 years) and 24.2% of tumors were diagnosed in women. All tumors were high grade and 5 were stage I, 20 were stage II, and 34 stage III-IV (among HER2 gastric tumors, 11 were stage II and 21 stage III-IV) according to the TNM staging system. HER2 staining was performed at the diagnosis following the established protocols [27] and 31 were considered as HER2-positive. Immunohistochemical and clinical data of HER2 breast and gastric tumors are provided in Supplementary Table 1. This study was performed following standard ethical procedures of the Spanish regulation (Ley de Investigación Orgánica Biomédica, 14 July 2007) and was approved by the ethic committees of the MD Anderson Cancer Center Madrid, Madrid, Spain.

### Cell culture and *in vitro* assays

HCC1954 (derived from a human invasive ductal breast carcinoma), NCI-N87 (human gastric adenocarcinoma) and HEK293T cell lines were obtained from the American Type Cell Culture (ATCC) and OE19 (human esophageal adenocarcinoma) cell line from the Deutsche Sammlung von Mikroorganismen und Zellkulturen (DSMZ). Cells were cultured following the supplier conditions. Cells were authenticated by STR-profiling according to ATCC or DSMZ guidelines. To generate lapatinib resistant (named LR) cell lines, both HCC1954 and OE19 parental cells were cultured for up to 6 months in the presence of increasing concentrations of lapatinib from 0.2 μM up to 2 μM (HCC1954 cells, which corresponds to IC50) or 1.5 μM (OE19, IC70). Resistant status was analyzed by cell viability assays using AlamarBlue (Bio-Rad), according to the manufacturer’s protocol. Then, HCC1954 LR and OE19 LR cells were cultured in the continuous presence of 2 μM and 1.5 μM lapatinib, respectively. In parallel, HCC1954 and OE19 control cells (C) were generated by chronic treatment with the vehicle DMSO (same amount as the corresponding lapatinib-resistant cells). For the *in vivo* studies with mice, mCherry-luc transduced HCC1954 cells previously generated [14] were used. Cell viability assays, biochemical studies, immunofluorescence, and confocal imaging techniques were performed as described in Supplementary Material and Methods.

### Animal *in vivo* studies

All the experimental procedures with mice were approved by the internal ethical research and animal welfare committee (IDIBELL and IIB, UAM), and by the Local Authorities (Generalitat Catalana, B-9900010 and Comunidad de Madrid, PROEX 235.6/20, respectively). They complied with the European Union (Directive 2010/63/UE) and Spanish Government guidelines (Real Decreto 53/20133). Furthermore, zebrafish studies (AB strain, *Danio rerio*) were performed with the agreement of the Bioethics Committee for animal experimentation of the University of Santiago of Compostela (CEEA-LU), REGA code: ES270280346401. The detailed description of the different *in vivo* experiments is provided in Supplementary Material and Methods.

### Statistics and reproducibility

The Chi-square contingency test with Yates’s correction, or Fisher’s exact test, was used to determine the statistical significance of the relationships between immunohistochemical and clinico-pathological features. GraphPad Prism software was used for graphic representation and statistical analysis. Error bars represent the mean ± s.e.m of at least three independent experiments. Data were tested for normality, and paired sets of data were compared using unpaired Student's t-test (two-tailed).

## Results

### GSDMB upregulation in response to anti-HER2 therapies associates with drug resistance

**Fig. 1:**
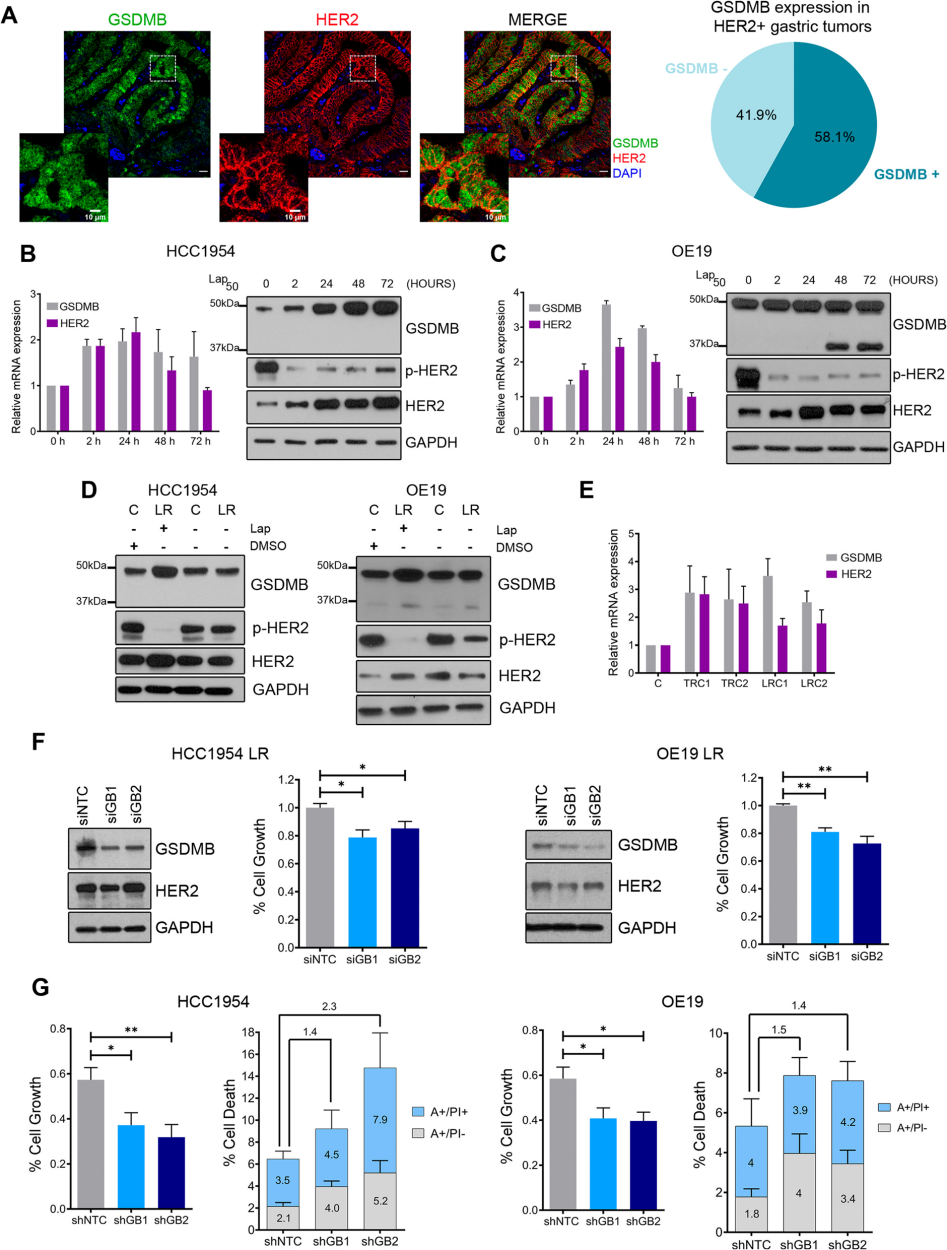
GSDMB is induced in response to anti-HER2 therapies, and its silencing increases the sensitive to lapatinib treatment. **A** Representative immunofluorescence images of GSDMB and HER2 expression in GSDMB/HER2-positive gastric carcinomas. Scale bar, 20 µm. Nuclei were counterstained with DAPI. Representative pie chart of GSDMB expression statistics in HER2 + gastric carcinomas (see Supplementary Table 1 for extra clinical features and statistics). **B-C** Relative mRNA (left) and protein levels (right) of GSDMB and HER2 in HCC1954 (**B**) and OE19 (**C**) cells treated with IC50 of lapatinib (2 µM and 0.7 µM, respectively) at indicated time points. **D** GSDMB and HER2 protein levels in lapatinib resistant HCC1954 and OE19 cells (LR) and their corresponding control cells (**C**) treated chronically with lapatinib and DMSO, respectively, and after ten days of drug removal. Study of the different cytotoxic effect of the chronic lapatinib treatment in these cells was analyzed by cell viability assays. **E** Relative mRNA levels of *GSDMB* and *HER2* in trastuzumab (TRC1 and TRC2) and lapatinib (LRC1 and LRC1) resistant tumors, compared to the parental tumor (**C**) derived from a HER2 breast cancer PDXs. **F** GSDMB expression was reduced in HCC1954 LR (left) and OE19 LR cells (right) by two specific siRNAs (siGB1 and siGB2), in comparison with the control (siNTC). Cytotoxic effect of the presence of lapatinib (2 µM and 1.5 µM, respectively) in GSDMB-siRNAs-silenced cells HCC1954 LR (left) and OE19 LR (right) cells was assessed by cell viability assays. **G** Cytotoxic effect after 72 h treatment with IC50 of lapatinib (2 µM and 0.7 µM, respectively) in GSDMB-shRNAs-silenced cells HCC1954 and OE19 (right) cells was assessed by cell growth (viability assays, left panel) and death (Annexin V FITC and PI, right panel). Annexin V-FITC positive cells alone (A + /PI-) and Annexin V-FITC and PI doubled stained (A + /PI +) were defined as apoptotic cells. The number over the bars indicate the ratio of cell death relative to the shNTC condition. Statistical significance was determined by two-tailed unpaired *t*-test (*P < 0.05; **P < 0.01). Data are shown as the mean ± s.e.m. Three independent experiments with similar results were performed. In (**B**, **C**, **E**), gene expression was normalized to the mRNA levels of *GAPDH*. A, Annexin V; PI, propidium iodide. NTC, non-targeting control. DMSO, dimethyl sulfoxide. Lap, lapatinib

Our previous work proved that GSDMB over-expression is a maker of poor prognosis associated with trastuzumab resistance in HER2 breast carcinoma patients in both neoadjuvant and adjuvant treatment settings [12, 14]. Moreover, high levels of GSDMB decrease sensitivity to trastuzumab *in vitro* in HCC1954 and SKBR3 cells, and its expression increases during the acquisition of trastuzumab resistance in HER2 + breast cancer PDX models [12]. Here, to address whether GSDMB also plays a role in the clinical behavior of gastric tumors, we first observed that strong cytoplasmic and nuclear GSDMB staining (Fig. 1A and Supplementary Table 1) associated statistically with HER2 positive status (GSDMB high expression in 18/31 (58.1%) of HER2-positive and 8/28 (29%) of HER2-negative tumors; *p* = 0.023). In HER2 gastric carcinomas, alike HER2 breast tumors [12], GSDMB over-expression associates with relapse (*p* = 0.060, Supplementary Table 2), thus supporting the relationship between high levels of GSDMB and poor prognosis in gastric tumors. Next, to test if in HER2 breast and gastroesophageal cancers GSDMB is functionally involved in regulating drug response/resistance also to the HER2 tyrosine kinase inhibitor lapatinib, we used three HER2 + cancer cell lines that endogenously express GSDMB, HCC1954 (breast cancer), OE19 (esophageal) and NCI-N87 (gastric). First, we treated these cells for different time points (up to 72 h) with their corresponding IC50 of lapatinib (Fig. 1B-C and Supplementary Fig. 1A). Additionally, for comparison, trastuzumab treatment was carried out only in OE19 and NCI-N87 cells (Supplementary Fig. 1B-C) since HCC1954 cells are intrinsically highly resistant to this drug [14, 28]. Both lapatinib (Fig. 1B-C and Supplementary Fig. 1A) and trastuzumab (Supplementary Fig. 1B-C) provoke a sharp induction of GSDMB mRNA, which peaked at 24–48 h, in all tested models. HER2 upregulation was also detected as previously reported [29]. At the protein level, while GSDMB was strongly upregulated by lapatinib in HCC1954 cells, the total amount of GSDMB protein was not clearly increased in gastroesophageal cancer cells (OE19 and N87) neither after lapatinib nor trastuzumab treatment. This is due to the appearance of a processed form of GSDMB protein (p37) (Fig. 1B-C and Supplementary Fig. 1A-C) at the latest treatment time points, that corresponds to the previously identified C-terminal cleavage product generated by apoptotic caspases-3/6/7 [30] (Supplementary Fig. 1D). GSDMB processing by caspases-3/6/7 generates N- (p10) and C-terminal (p37) fragments [30] that do not have an effect on cell death induction [6].

Next, to assess further if GSDMB induction correlates with acquired resistance to HER2-targeted therapies, we generated HCC1954 and OE19 cells long-term resistant (LR) to high doses (> IC50) of lapatinib. In both models, again, we observed an upregulation of GSDMB and HER2, and as expected, a strong decrease in HER2 phosphorylation [29], compared to their respective control cells (chronically grown with high doses of the vehicle DMSO) (Fig. 1D). Interestingly, GSDMB upregulation was dependent on lapatinib presence since drug removal for 10 days restored GSDMB protein levels (Fig. 1D). Moreover, as a complementary model, we used primary cultures from previously generated HER2 + breast cancer PDXs [31]. Thereby, compared to the parental cell line (C), which is sensitive to trastuzumab, the *in vitro* resistant clones to trastuzumab (TRC1 and TRC2) or lapatinib (LRC1 and LRC2) showed a significant GSDMB upregulation (Fig. 1E). These *in vitro* results in various cell models (breast and gastric cancer cell lines, breast PDX primary culture), together with our published observations *in vivo* with PDXs and HER2 breast carcinoma patients [12], suggest that the GSDMB over-expression would induce resistance to trastuzumab as well as lapatinib. Since TKIs are mainly utilized in trastuzumab pretreated patients [17] (indeed HCC1954 cells are intrinsically resistant to trastuzumab [14, 28]) and there is a need to understand the molecular mechanisms that underlies the TKI derived resistance, we selected lapatinib as the suitable (second line) anti-HER2 therapy to further decipher the connection between GSDMB and drug resistance.

To study if GSDMB is functionally involved in the early response and long-term resistance to lapatinib, we silenced GSDMB in OE19 and HCC1954 cells by shRNA (two different sequences shGB1 and shGB2), as previously reported [13, 14]. These shRNAs robustly decrease GSDMB expression at the protein (Supplementary Fig. 1E) and mRNA levels (all four GSDMB isoforms that translate to protein) in OE19 (Supplementary Fig. 1F) and HCC1954 cells [14], and do not target other GSDM genes (Supplementary Fig. 1G). Furthermore, in the resistant HCC1954 LR and OE19 LR cells, GSDMB expression was transiently reduced by two different GSDMB-specific siRNAs (siGB1/2, Fig. 1F). Stable GSDMB-silencing by lentiviral shRNA transduction could not be obtained in these models because they exhibited intrinsic resistance to the selection antibiotic puromycin.

GSDMB-shRNA-silenced HCC1954 and OE19 cells were significantly more sensitive to lapatinib treatment, as they exhibited an important reduction in cell viability and an increase in cell death compared to shNTC control cells (Fig. 1G). Likewise, in the LR models with siGSDMB, we found a slight but statistically significant decrease (around 20%) in cell viability in the presence of lapatinib (Fig. 1F).

### GSDMB increases pro-survival autophagy in response to lapatinib treatment

GSDMB silencing did not affect the levels of HER2 receptor in any of our cell models (Supplementary Fig. 2A), suggesting that GSDMB does not promote response/resistance to lapatinib through direct modulation of HER2 quantity. Therefore, to decipher the molecular mechanism by which GSDMB modulates lapatinib response, we focused on autophagy, as this process has been demonstrated to act as a resistance mechanism to anti-HER2 therapies both *in vivo* and *in vitro* [22]. Autophagy induction during tumor progression can lead to either survival (pro-tumor) or cell death (anti-tumor) depending on the stimulus and the cellular context [21]. Hence, we first tested in HCC1954 and OE19 parental cells if lapatinib treatment induced autophagy with survival or death consequences (Supplementary Fig. 2B-E). In both cell lines, lapatinib treatment induced autophagic response at 24–48 h, measured by the increase in the levels of LC3B-II (Supplementary Fig. 2B). Importantly, this autophagy is protective since blocking autophagic flux with chloroquine (CQ), which affects the completion of the latter stages of autophagy [32], significantly increased the cytotoxicity of lapatinib (Supplementary Fig. 2C). Similarly, blocking the formation of autophagosomes [33] by an ATG5-specific siRNA (Supplementary Fig. 2D) enhanced the effect on cell viability of lapatinib (Supplementary Fig. 2E).

**Fig. 2:**
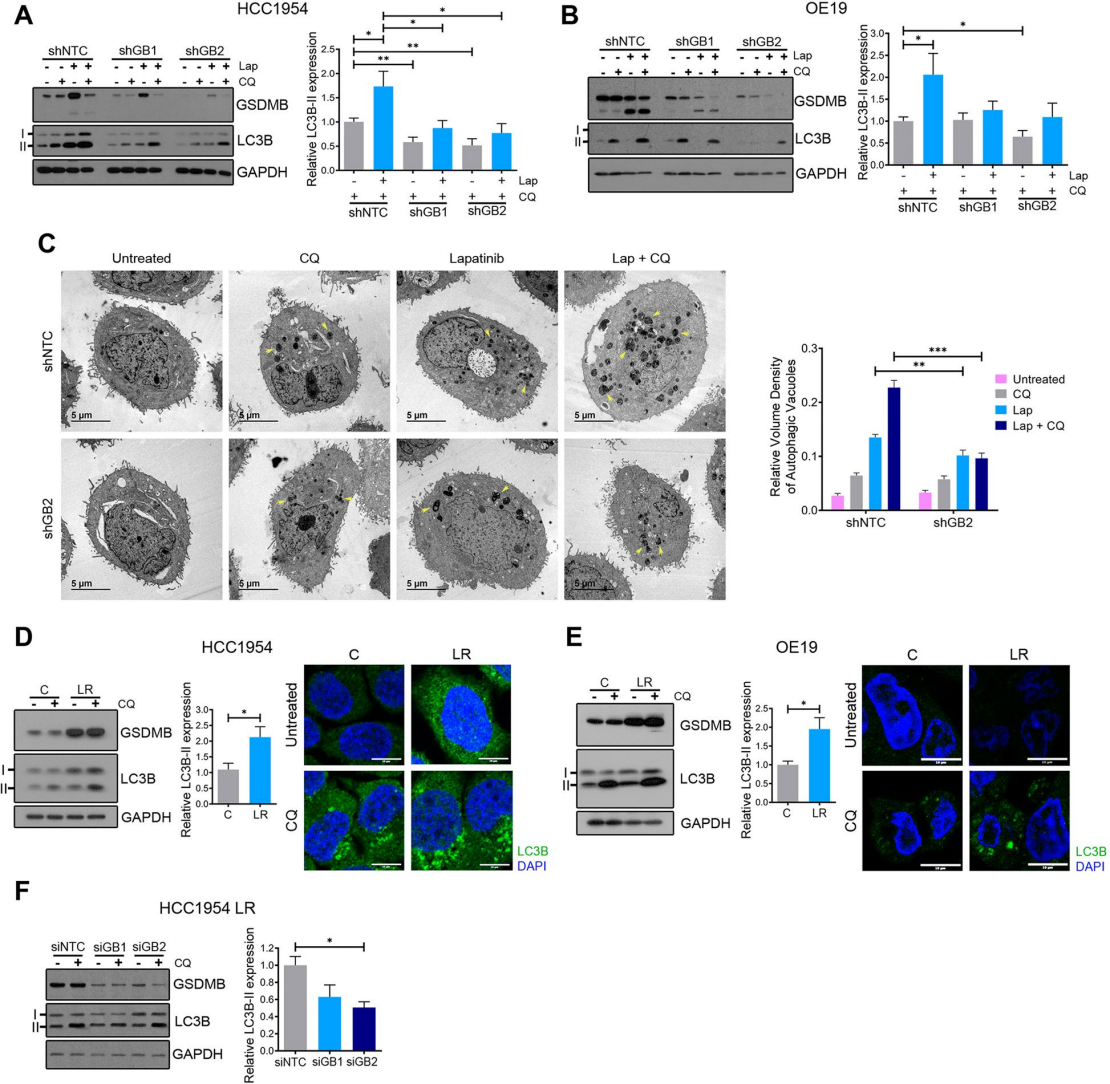
GSDMB-high cells show an increased autophagic flux in response to lapatinib. **A-B** GSDMB and LC3B protein levels in shNTC, shGB1 and shGB2 HCC1954 (**A**) and OE19 (**B**) cells treated with lapatinib (Lap, 2 µM and 0.7 µM, respectively) and/or CQ (10 µM and 50 µM, respectively) for 72 h. Quantification of the relative LC3B-II expression was conducted as described before [34, 35]. **C** Representative transmission electron microscopy images of shNTC and shGB2 HCC1954 cells treated with the treatment regimens indicated in (**A**). Quantification of the relative volume density of autophagic vacuoles is shown on the right. At least 25 cells were analyzed per experimental condition. **D-E** Western blot analysis of GSDMB and LC3B (left panels) in HCC1954 LR (**D**) and OE19 LR (**E**) cells and their respective controls (**C**) treated with or without CQ (10 µM and 50 µM, respectively) for 72 h. LC3B expression (green) analysis by confocal microscopy (right panels) in HCC1954 LR (**D**) and OE19 LR (**E**) cells and their controls (**C**) treated with or without CQ at the concentrations indicated in (**A-B**). Representative confocal microscopy images were shown, scale bar, 10 µm. Nuclei were counterstained with DAPI. **F** GSDMB and LC3B protein levels in GSDMB-siRNA-silenced HCC1954 LR cells treated with or without 10 µM CQ for 72 h. Quantification of LC3B-II expression (showed on the right of panels, **A-B, D-F**) was carried out by densitometric scanning and normalized to GAPDH expression following previous methods [34, 35]. Statistical significance was determined by two-tailed unpaired *t*-test (*P < 0.05; **P < 0.01). Data are shown as the mean ± s.e.m. Three independent experiments with similar results were performed. NTC, non-targeting control. LR, Lapatinib resistant cells. CQ, chloroquine. Lap, lapatinib

Next, we assessed whether cells with high or low GSDMB expression could have different endogenous autophagic responses by measuring autophagic flux as the accumulation of the lipidated LC3B (LC3B-II) form in western blots [34, 35]. In this regard, we analyzed LC3B-II turnover in the presence and absence of lysosomal degradation using CQ. Therefore, higher LC3-II levels were observed in GSDMB-expressing (shNTC) HCC1954 and OE19 cells in comparison with GSDMB-silenced cells in basal autophagy, (Fig. 2A-B**,** see grey bars) and this effect was significantly exacerbated upon autophagy activation with lapatinib (Fig. 2A-B**,** see blue bars). It should be noted that due to the high efficacy of the combined treatment, the enhanced cell death resulted in an overall degradation of proteins, including GSDMB and GAPDH (Fig. 2A-B). Besides, a significant increase in the relative volume density of autophagic vacuoles was observed by transmission electron microscopy in GSDMB-expressing HCC1954 cells, compared to GSDMB-silenced cells, after the treatment with lapatinib and its combination with CQ (Fig. 2C). In the same way, HCC1954 LR and OE19 LR cells, which express high levels of GSDMB, exhibit increased LC3B-II accumulation by western blot and LC3B-II puncta by confocal imaging (Fig. 2D-E) compared to their respective control cells. Thus, these results suggest that high GSDMB expression somehow increases the intrinsic autophagic response to cell stress. Accordingly, we confirmed that the autophagic flux induced by lapatinib (Fig. 2A-C), or by serum starvation (Supplementary Fig. 3A-B) is reduced in GSDMB-silenced cells compared to control lines. Moreover, in HCC1954 LR cells grown in the presence of high doses of lapatinib, the reduction of GSDMB expression by siRNAs also diminishes the autophagic flux (Fig. 2F). Furthermore, we discarded that this observed autophagic response involved selective autophagy subtypes such as mitophagy (Supplementary Fig. 4A), aggrephagy (Supplementary Fig. 4B) or lipophagy (Supplementary Fig. 4C).

**Fig. 3:**
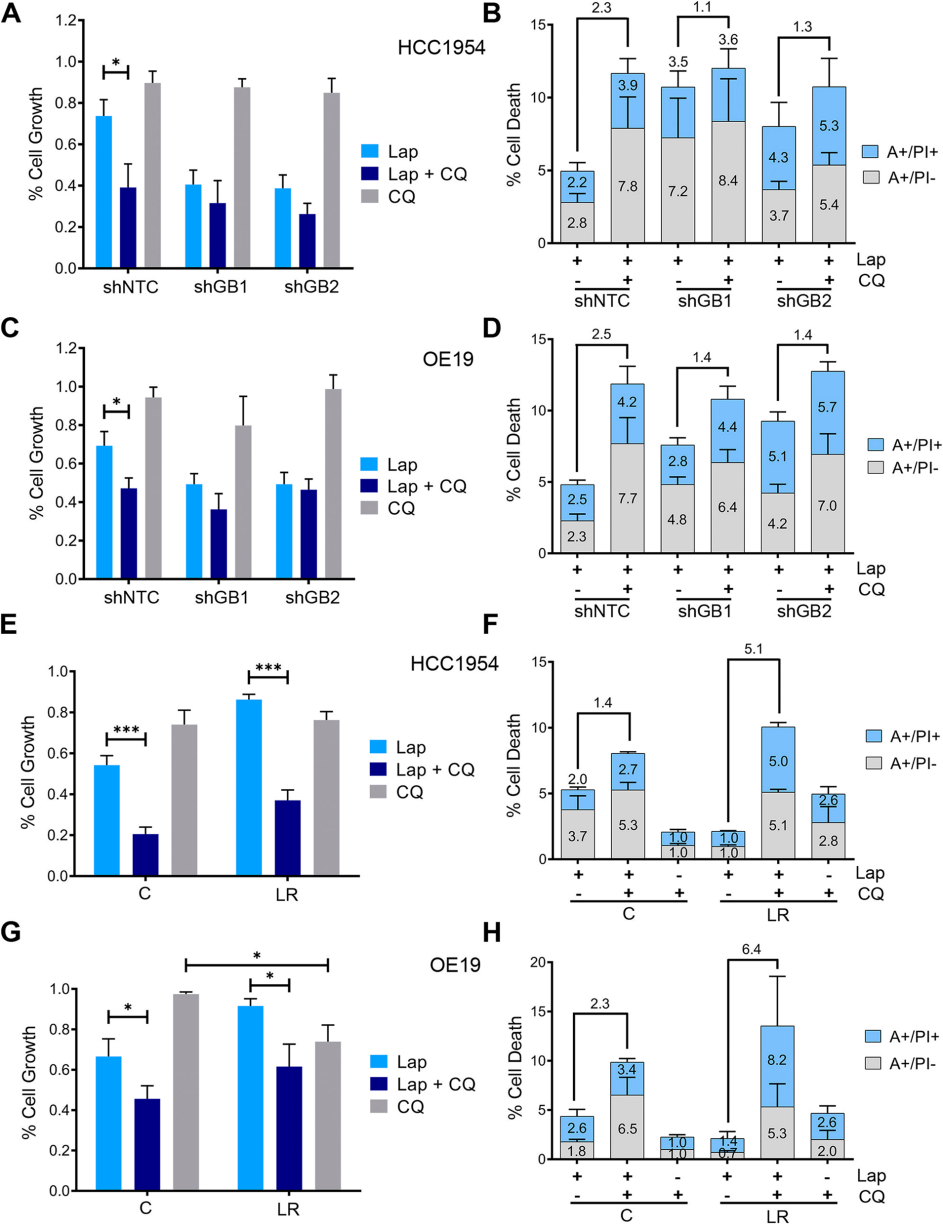
GSDMB-high cells are significantly more sensitive to the combination of lapatinib plus chloroquine. **A-D** The cytotoxic effect of the treatment with lapatinib and/or chloroquine in shNTC, shGB1 and shGB2 HCC1954 (**A, B**) and OE19 (**C, D**) cells was evaluated by cell viability assays and Annexin V-FITC plus PI. **E–H** The outcome in terms of cell viability and apoptosis of the different treatment regimens was analyzed in HCC1954 LR (**E–F**) and OE19 LR (**G-H**) cells compared to their corresponding control cells **C**. Statistical significance was determined by two-tailed unpaired *t*-test (*P < 0.05; **P < 0.01; ***P < 0.001). Data are shown as the mean ± s.e.m. Annexin V-FITC positive cells alone (A + /PI-) and Annexin V-FITC and PI doubled stained (A + /PI +) were defined as apoptotic cells. The number over the bars indicate the fold increase in cell death between the indicated conditions (**B, D, F, H**). Three independent experiments with similar results were performed. A, Annexin V; PI, propidium iodide; CQ, chloroquine. NTC, non-targeting control. LR, Lapatinib resistant cells. Lap, lapatinib, CQ, chloroquine

Then, given that GSDMB-high cells exhibit increased autophagic lapatinib response and that this process promotes survival in our cell models (Supplementary Fig. 2), we postulated that GSDMB-high cells could be particularly sensitive to the combination of lapatinib with autophagy inhibitors. Indeed, the autophagy blockage with CQ in GSDMB-high (shNTC) HCC1954 and OE19 cells treated with lapatinib produces a significant reduction in the cell viability (Fig. 3A-C) as well as an increase in cell death rates (Fig. 3B-D) compared to lapatinib alone. In contrast, no such dramatic effect was observed in the GSDMB-silenced cells. Importantly, in HCC1954 LR and OE19 LR cells, which over-express GSDMB, the addition of CQ strongly increases the cytotoxic effect of lapatinib, and thus revert their resistance to this anti-HER2 drug (Fig. 3E-H). Consistent with these results, the autophagy blockage by siATG5 confirmed the increased sensitivity to lapatinib in GSDMB-high cells in all cell models (Supplementary Fig. S5a-d). It should be noted that particularly in OE19 LR cells, which show the highest GSDMB levels, the autophagy inhibition alone (either by CQ or siATG5) has a significant effect on cell viability (Fig. 3G and Supplementary Fig. 5D), supporting that after long-term challenge with this anti-HER2 therapy the subsistence of these cells mostly relies on the endogenous pro-survival autophagic process.

Taken together, these results confirm that GSDMB enhances the pro-survival autophagy in response to lapatinib in HER2 + breast and gastric cancer cells, and thus GSDMB-over-expressing cells are more sensitive to the addition of autophagy inhibitors both in the early response to HER2-targeted treatment and in drug resistant cells.

### Autophagy inhibition enhances lapatinib efficacy *in vivo* specifically in GSDMB-expressing breast cancer cells

**Fig. 4:**
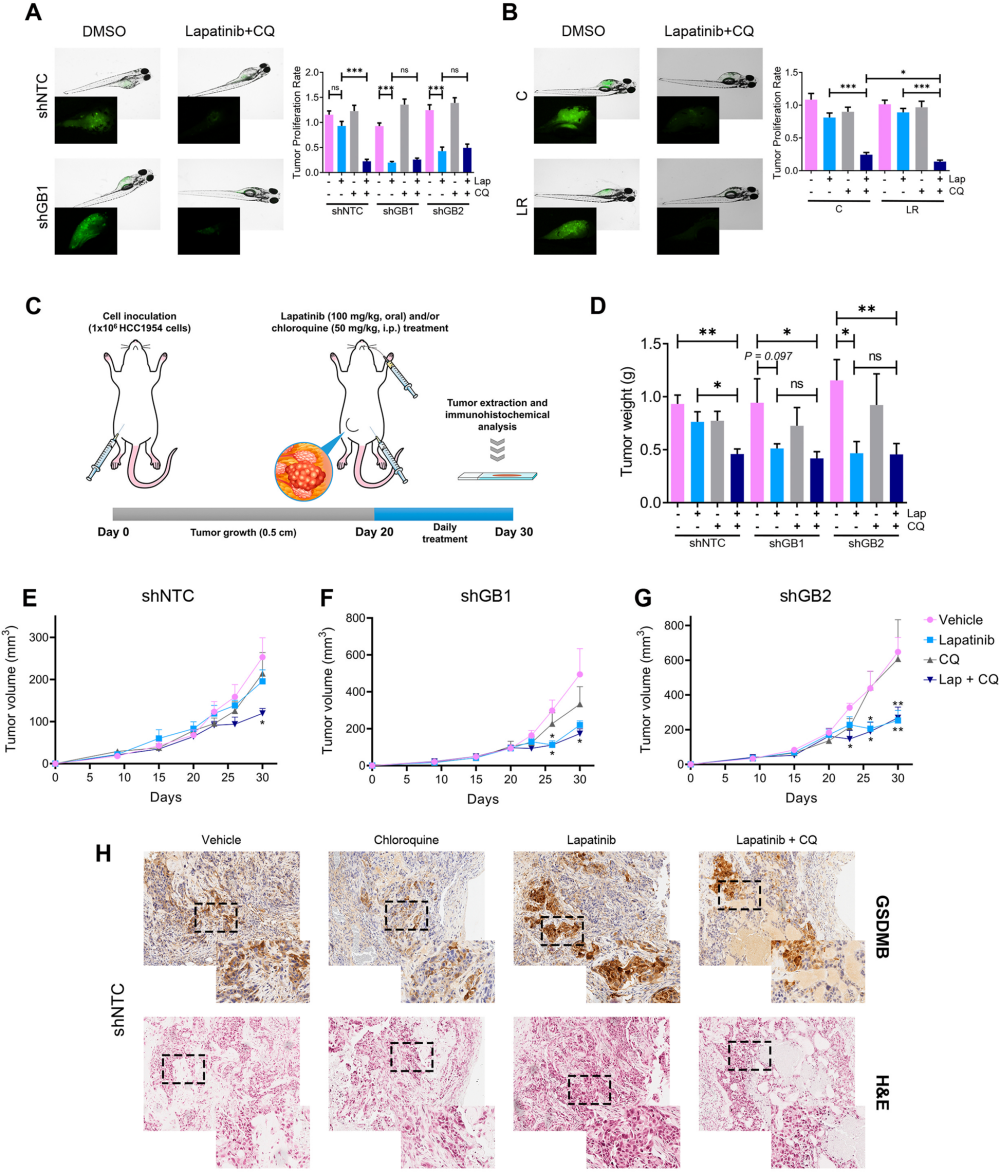
Autophagy blockade with chloroquine improves lapatinib efficacy *in vivo* in zebrafish and mouse xenografts of GSDMB-expressing tumors. **A-B** Representative fluorescence stereomicroscope images (left panel) of GFP expressing control (shNTC), and GSDMB-silenced (shGB1) HCC1954 xenografts (**A**) or HCC1954 LR and control (**C**) tumors (**B**) treated with the LC50 of lapatinib and chloroquine (35,1 mM and 116,4 mM, respectively). Insets represent an augmented image of GFP-positive tumor. Tumor proliferation rate (right panel) of the different HCC1954 xenografts was analyzed by measuring the fluorescence intensity ratio (48 hpt/0 hpt). Statistical significance was determined by two-tailed unpaired *t*-test. At least, *n* = 20 per each indicated condition. **C** Experimental design of the mouse xenograft model (*n* = 5 per condition). Mice were inoculated with either HCC1954-mCherry-luc control (shNTC), or GSDMB-silenced cells (shGB1 and shGB2) and treated with lapatinib (100 mg/kg, orally, once daily), CQ (50 mg/kg, intraperitoneally, once daily), or a combination of both (lapatinib + CQ). An aqueous solution containing 0.1% Tween 80 and 0.5% Hypromellose was used as vehicle. The experiment was performed for 30 days, according to the approved protocol and conditions of animal research (detailed in Supplementary Methods). **D-G** Quantification of the tumor weight (**D**) tumor volume evolution of shNTC (**E**), shGB1 (**F**) and shGB2 (**G**) HCC1954 xenografts, treated with the indicated regimens. Statistical significance was determined by multiple unpaired *t*-test – comparing vehicle with each of the other conditions at every time point. **H** Representative images of GSDMB immunohistochemical analysis and hematoxylin and eosin staining in shNTC tumors, treated with the different therapeutic strategies indicated in (**C**). Immunohistochemical images were taken on 10X and 40X (insets) magnification. (*P < 0.05; **P < 0.01; ***P < 0.001; ns, nonsignificant). Data are shown as the mean ± s.e.m. LC50, 50% lethal concentration; hpt, hours post-treatment. NTC, non-targeting control. LR, Lapatinib resistant cells, CQ, chloroquine. Lap, lapatinib

Next, to validate if the combination of lapatinib and CQ on HER2/GSDMB positive tumors would be an effective therapeutic approach, we assayed its functional effect on tumor growth *in vivo* using two different preclinical models, zebrafish, and mice (Fig. 4). In zebrafish, which allows testing the drug response in a large number of biological replicates [36], we first calculated the acute dose-dependent toxicity of both lapatinib and CQ by analyzing the embryo mortality (Supplementary Table 3). Next, the different HCC1954 models mentioned above (shNTC, shGB1/2 as well as LR and the corresponding control cells) that stably express GFP were inoculated into the yolk sac of zebrafish embryos and treated with either lapatinib, CQ or the combination of both (Fig. 4A-B and Supplementary Fig. 6A-B). As expected, lapatinib treatment produced a significant reduction in cancer cell growth of GSDMB-silenced tumors (shGB1 and shGB2), but not in HCC1954 shNTC (Fig. 4A). Remarkably, autophagy blockage by CQ provoked an increase in the effect of lapatinib on tumor growth reduction (*p* < 0.001) only in GSDMB-expressing (shNTC) tumors, compared to lapatinib alone, while no such effect was observed in GSDMB-silenced (shGB1, shGB2) tumors. Furthermore, similar results were found on lapatinib resistant cells (LR), where the combined treatment (CQ plus lapatinib) practically abolished the resistant phenotype of HCC1954 LR cells (Fig. 4B and Supplementary Fig. 6B). These findings support that the combination therapy was effective specifically in high GSDMB-expressing tumors.

Subsequently, to validate these results, we performed similar treatment experiments in mice bearing orthotopically injected HCC1954 (shNTC, shGB1 and shGB2) breast cancer xenografts (Fig. 4C-H and Supplementary Fig. 7). Once again, while lapatinib alone mainly decreased tumor weight and volume in GSDMB-silenced tumors (Fig. 4D,F,G and Supplementary Fig. 7A), the addition of CQ enhanced the effect of lapatinib on the reduction of tumor growth exclusively in GSDMB-expressing (shNTC) tumors (Fig. 4D-E). The therapeutic response was independent of cell proliferation since no differences in PCNA immunohistochemical expression were detected in any of the experimental conditions (Supplementary Fig. 7B). As observed *in vitro*, GSDMB was also up-regulated *in vivo* in GSDMB-expressing tumors (shNTC) by lapatinib, noting an increase in the necrotic areas in the case of the combined treatment (Fig. 4H and Supplementary Fig. 7B), and again it was associated with a reduced response to the treatment compared to GSDMB-silenced tumors (Fig. 4D,F,G and Supplementary Fig. 7C). These *in vivo* data prove that the autophagy blockage is essential to improve the anti-HER2 therapy response in HER2/GSDMB tumors and reinforce the role of GSDMB in the promotion of pro-survival autophagy as a resistance mechanism to these therapies.

### GSDMB acts as an autophagy adaptor inducing Rab7 activation with a predictive value to anti-HER2 therapies

**Fig. 5:**
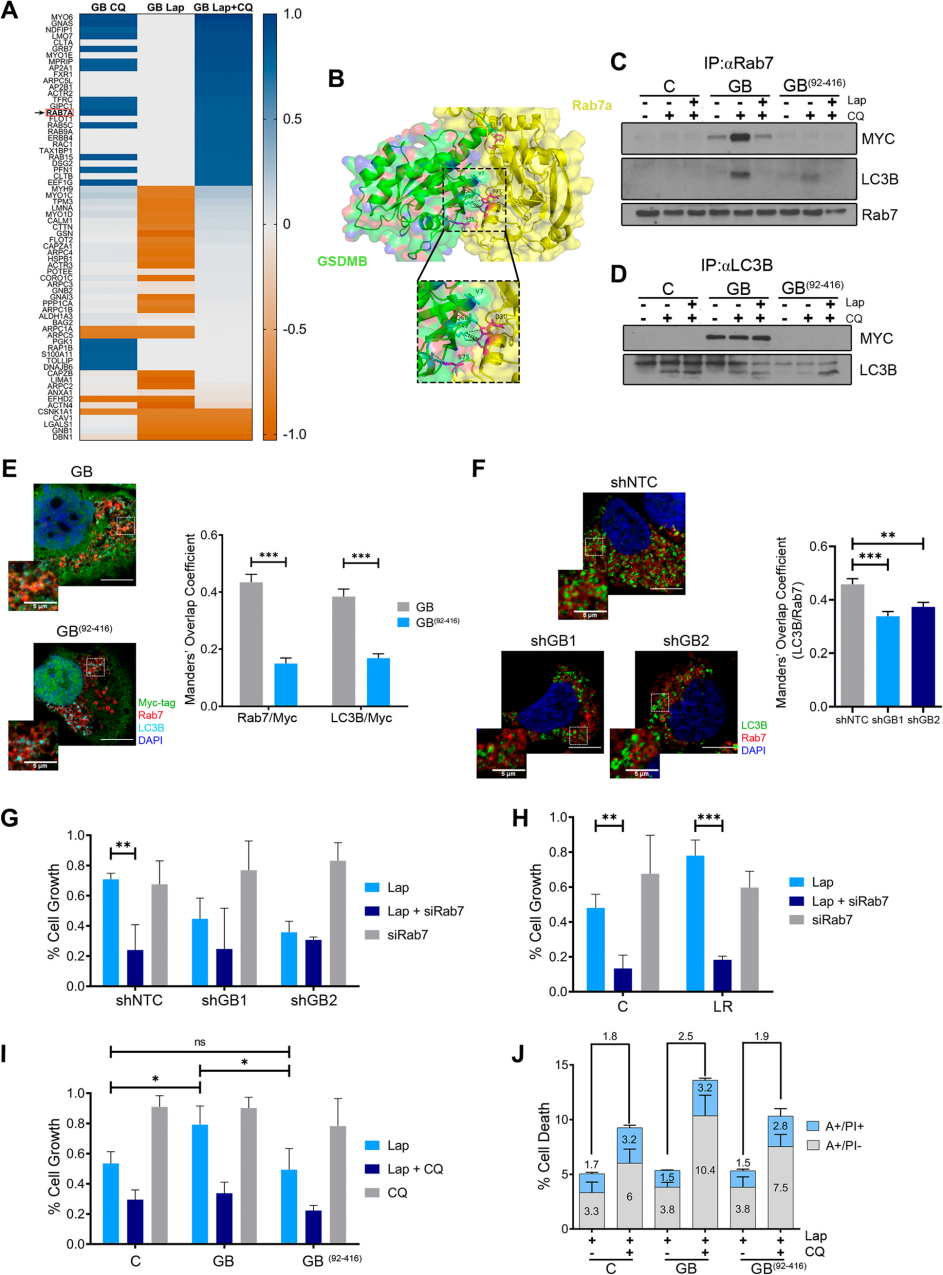
GSDMB modulates autophagic response through Rab7 and LC3B interaction. **A** Heatmap representation of the mass spectrometry analysis performed on HCC1954 GB cells treated with the different treatment regimens, after co-immunoprecipitation assay using a Myc-tag antibody. Normalization to untreated cells was performed. Color key indicates protein expression score: dark blue: highest; dark orange: lowest. **B** In silico protein interaction prediction using the InterEvDock2 and PPCheck web servers identifies a potential interaction between Rab7a (yellow) and the N-terminal domain of GSDMB (green). Inset magnifies the predicting interacting region (pink). 3D structures were obtained from Uniprot data bases (Q8TAX9: GSDMB and P51149: Rab7a). **C-D** Co-immunoprecipitation assay of Rab7-GSDMB (**C**) and LC3B-GSDMB (**D**) interaction after CQ and lapatinib plus CQ treatment in HCC1954 cells exogenously expressing full length GSDMB (GB), a construct lacking the N-terminal domain (GB^(92–416)^) or control (C; empty vector) cells. **E** Representative images of the colocalization between Rab7 (red), LC3B (cyan) and GSDMB-Myc-tag (green) by confocal microscopy in HCC1954 GB and GB^(92–416)^ cells after lapatinib (2 µM) plus CQ (10 µM) treatment. Nuclei were counterstained with DAPI. Quantification of the Manders’ Overlap Coefficient (Rab7 overlapping Myc-tag and LC3B overlapping Myc-tag) is shown on the right. Five independent experiments were performed obtaining at least 60 cells, per experimental condition. Scale bar, 10 µm. **F** Representative images of the colocalization between Rab7 (red) and LC3B (green) by confocal microscopy in shNTC, shGB1 and shGB2 HCC1954 cells after lapatinib (2 µM) plus CQ (10 µM) treatment. Nuclei were counterstained with DAPI. Quantification of the Manders’ Overlap Coefficient (LC3B overlapping Rab7) is shown on the right. Five independent experiments were performed obtaining at least 60 cells, per experimental condition. Scale bar, 10 µm. **G-H** The cytotoxic effect of the treatment with lapatinib plus/or siRab7a-silencing was evaluated by cell viability assays in shNTC, shGB1 and shGB2 HCC1954 (**G**) and HCC1954 LR (**H**) cells. **I-J** Comparative cytotoxic effect by cell viability assays (**I**) and Annexin V-FITC plus PI (**J**) in HCC1954 C, GB and GB^(92–416)^ cells after 72 h treatment with lapatinib (2 µM) and/or CQ (10 µM). Annexin V-FITC positive cells alone (A + /PI-) and Annexin V-FITC and PI doubled stained (A + /PI +) were defined as apoptotic cells. Statistical significance was determined by two-tailed unpaired *t*-test (*P < 0.05; **P < 0.01; ***P < 0.001; ns, nonsignificant). Data are shown as the mean ± s.e.m. In (**E-J**), three independent experiments with similar results were performed. Lap, lapatinib, CQ, chloroquine

To unravel further the role of GSDMB in autophagy, HCC1954 cells exogenously over-expressing myc-tagged full length GSDMB (GB) after treatment with lapatinib, CQ, or both drugs were immunoprecipitated with a Myc-tag antibody followed by mass-spectrometry studies. Among potential GSDMB interacting cancer and autophagy proteins, some Rab GTPases were found (Rab5C, Rab7A, Rab9A and Rab15, Fig. 5A and Supplementary Table 4). These have been implicated in different steps of the autophagy process [37]; especially, Rab7 which participates in the autophagosome-lysosome fusion, the autolysosome maturation and transport [25, 38] and has shown a pivotal role in resistance to chemotherapeutic agents [39]. Rab7a was found with a higher score both in CQ and combined treatment in the mass-spectrometry assay compared to untreated cells (Fig. 5A). To identify the interaction region between GSDMB and Rab7a, an in silico prediction assay was carried out, revealing an energetically feasible GSDMB/Rab7a complex (Δ*G* = -2.73 kJ/mol), in which the most probable GSDMB residues involved in this interaction are distributed through its N-terminal region (amino acids V7, D30, F46, Q60 and E185 from GSDMB, Fig. 5B). Interestingly, we also found in the GSDMB N-terminal domain two putative LC3B-interacting region (LIR) motifs (“LIR1” corresponding to ^3^SVFEEI^8^ sequence and “LIR2” covering ^82^AEFQIL^87^ amino acids, Supplementary Fig. 8A). Despite LC3B was not detected in the proteomic analysis, we hypothesized that a GSDMB-Rab7a-LC3B multiprotein complex could occur. In fact, the HawkDock web server [40], predicted a highly possible GSDMB/Rab7a/LC3B complex (ΔG = -2.17 kJ/mol, Supplementary Fig. 8B). By contrast, a similar complex was not feasible when the rest of human GSDMs were analyzed (data not shown), supporting the idea that although other GSDMs have been previously implicated in autophagic processes [4, 41, 42], only GSDMB might regulate autophagy through Rab7a/LC3B interaction*.* Thus, to validate these results experimentally, we utilized HCC1954 parental cells with endogenous GSDMB levels (Control, C), cells with exogenous GSDMB overexpression (GB) and a model exogenously over-expressing the C-terminal (GB^92−416^) fragment produced by caspase 3/6/7 processing [30], which lacks both LIR motifs.

First, we noticed that neither GB nor GB^92−416^ over-expression significantly modifies Rab7 and LC3B endogenous levels (Supplementary Fig. 8C). In the co-immunoprecipitation assays, the interaction between GSDMB and Rab7 was detected only in HCC1954 GB cells but not in GB^92−416^ or control cells (Fig. 5C and Supplementary Fig. S8D). As expected, LC3B and Rab7 co-immunoprecipitation was mainly found after autophagy inhibition by CQ treatment in HCC1954 GB cells (Fig. 5C). Similar results were observed in the binding assays between GSDMB and LC3B (Fig. 5D and Supplementary Fig. 8D). Furthermore, by confocal microscopy, GSDMB colocalization with L3CB or Rab7 was significantly higher in GB than in GB^92−416^ expressing cells treated with lapatinib plus CQ (Fig. 5E and Supplementary Fig. 8E). Consistently, Rab7-LC3B colocalization was also significantly higher in GSDMB-expressing cells (shNTC) compared to shGB1/2 cells (Fig. 5F and Supplementary Fig. 8F). Besides, to prove the involvement of Rab7 in GSDMB-mediated resistance to anti-HER2 therapies, we silenced Rab7a (siRab7a) and confirmed the increased sensitivity to lapatinib especially in GSDMB-expressing HCC1954 cells (Fig. 5G and Supplementary Fig. 8G) and in HCC1954 LR cells (Fig. 5H). Together, these data indicate that the GSDMB N-terminal region comprising the amino acids 1–91 is necessary for the interaction with Rab7 and LC3B and for the subsequent autophagy induction mediated by GSDMB over-expression during lapatinib treatment. Supporting this idea, a significant cell growth increase was observed in HCC1954 GB cells compared to HCC1954 control or GB^92−416^ cells in response to lapatinib (Fig. 5I). Likewise, the cell death rate after the combination of lapatinib plus CQ was higher in HCC1954 GB cells than in the other conditions (Fig. 5J). Moreover, although the basal autophagy, measuring LC3B-II levels, induced by CQ treatment in HCC1954 GB, GB^92−416^ and control cells was similar (Supplementary Fig. 8C**,** see grey bars), the increase in autophagic flux by lapatinib plus CQ treatment was significantly stronger in HCC1954 GB cells, compared to control or GB^92−416^ cells (Supplementary Fig. 8C, compare blue and grey bars)**.** Together, the results support the key role of the GSDMB N-terminal region, particularly the first 91 residues, in the control of the autophagy response after anti-HER2 treatment.

In parallel, to validate our results in a GSDMB null biological context, thus avoiding the effects of low endogenous GSDMB expression in shGB models, we generated complete GSDMB-knockout cells by CRISPR/Cas9 (sgGB1). As expected, the autophagic flux induction in sgGB1 cells was indeed lower than in control cells (Supplementary Fig. 9A) and consequently the cytotoxic effect of lapatinib treatment was much higher in GSDMB-null cells (Supplementary Fig. 9B). In agreement with the results in the other cell models, after lapatinib plus CQ treatment the colocalization between LC3B and Rab7 was lower in GSDMB-knockout cells, confirming GSDMB involvement in the formation of an autophagic multiprotein complex (Supplementary Fig. 9C). Indeed, compared to shGB1/2 cells (Supplementary Fig. 8F), sgGB1 cells exhibited distinct Rab7 and LC3B staining patterns, being LC3B very diffuse and Rab7 rarely localized into vesicles (Supplementary Fig. 9C). This suggest that complete lack of GSDMB strongly decreases Rab7-mediated maturation of autophagosomes.

**Fig. 6:**
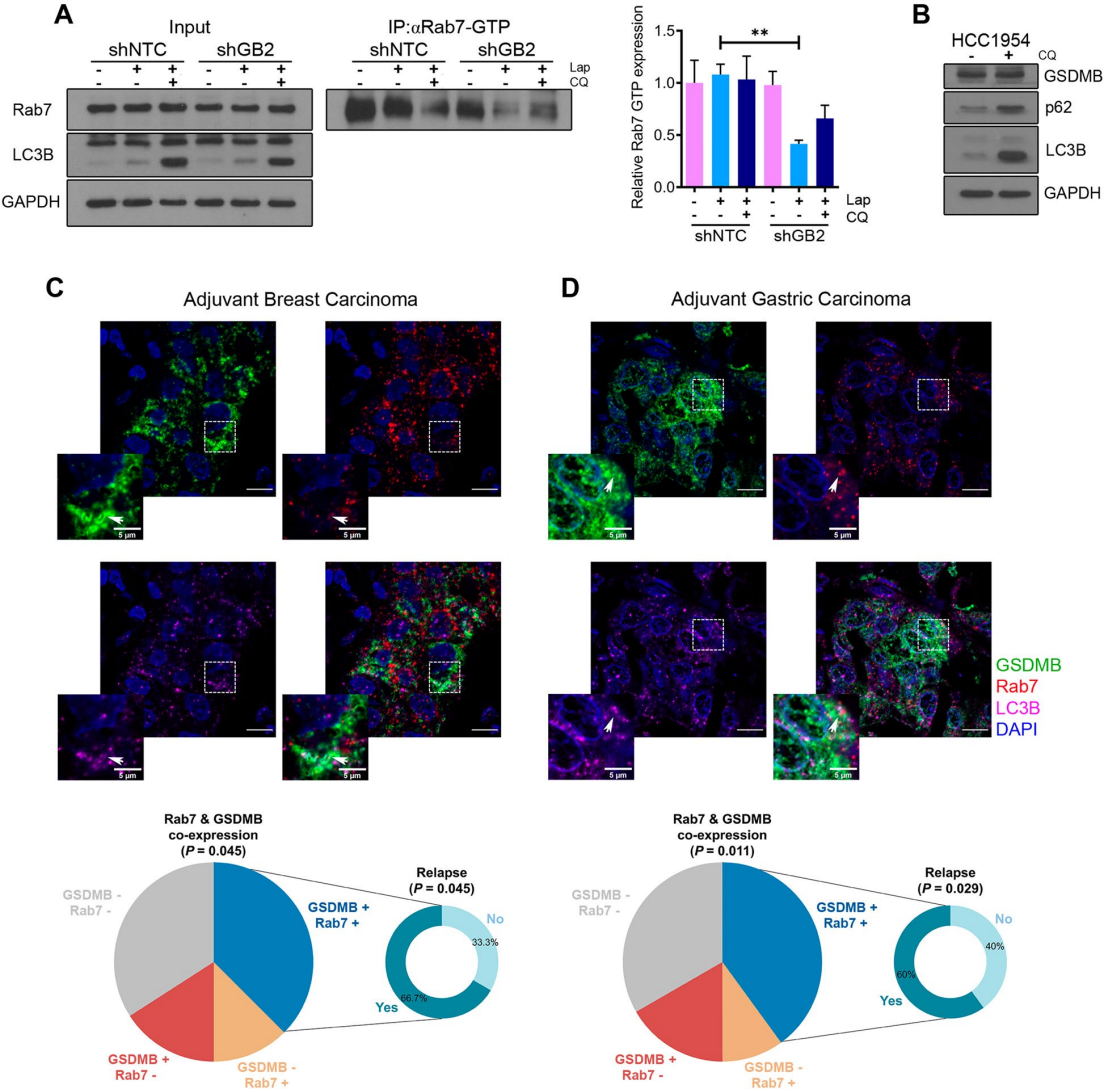
GSDMB expression promotes Rab7 activation in response to anti-HER2 drugs. **A** Co-immunoprecipitation assay of Rab7-GTP levels in shNTC and shGB2 HCC1954 cells after the treatment with lapatinib (2 µM) and the combined treatment. Statistical significance was determined by two-tailed unpaired *t*-test (**P < 0.01). Data are shown as the mean ± s.e.m. Three independent experiments with similar results were performed. **B** GSDMB, p62 and LC3B protein levels in HCC1954 cells treated for 72 h with or without chloroquine (10 µM). **C-D** Representative multiplex immunofluorescence images of GSDMB (green), Rab7 (red) and LC3B (deep red) co-expression in adjuvant breast (**C**) and gastric carcinomas (**D**). Scale bar, 20 µm. Nuclei were counterstained with DAPI. Representative pie charts of Rab7 and GSDMB co-expression statistics and its relationship with relapse in adjuvant breast (**C**) and gastric carcinomas (**D**) (see Supplementary Tables 1, 2 and 5 for extra clinical features and statistics). CQ, chloroquine. Lap, lapatinib

Therefore, to demonstrate the functional connection between GSDMB and Rab7 and the subsequent modulation of pro-survival autophagy we measured Rab7 activity (by co-immunoprecipitation assays with an anti-Rab7GTP antibody) in cells treated with lapatinib and the combined treatment (Fig. 6A). GSDMB-expressing cells showed higher Rab7-GTP levels after lapatinib or the combination treatment (*P* = 0.2195) compared to shGSDMB cells (Fig. 6A), thus demonstrating an important effect of GSDMB on Rab7 activity. Then, we hypothesized that GSDMB could act like an autophagy receptor or adaptor [43]. Both types of molecules bind Atg8 family proteins, like LC3B, but autophagy receptors, such as p62, control cargo selection and therefore, are degraded by autophagy [43]; thus, their levels increase when the autophagic flux is blocked. In contrast to p62 or LC3B, GSDMB does not accumulate upon autophagy blockage by CQ (Fig. 6B). In consequence, our data indicate that GSDMB would act as an autophagy adaptor in response to anti-HER2 therapies due to its ability to interact with LC3B located in the outer autophagosome membrane and regulates the autophagic machinery through Rab7 activation.

Finally, we aimed to validate our *in vitro* and *in vivo* results on clinical specimens of HER2 breast and gastric carcinomas. The immunohistochemical study of GSDMB, LC3B and Rab7 provided different pieces of important information. First, about 50% of HER2 breast and gastric tumors showed intense LC3B dotted (puncta) expression while Rab7 puncta was more frequent in gastric than breast carcinomas (Fig. 6C-D and Supplementary Table 1). Second, strong Rab7 or LC3B puncta was significantly more frequent in GSDMB over-expressing breast and in gastric tumors (Fig. 6C-D and Supplementary Table 2). Third, although GSDMB localization is usually diffuse cytoplasmic or nuclear [12, 14, 44], a concurrent dotted staining was observed for GSDMB, Rab7 and LC3B puncta within tumor cells (Fig. 6C-D). Four, and most notably, the GSDMB/Rab7 and GSDMB/LC3B puncta positive co-expression significantly correlated with relapse in HER2/GSDMB + breast and gastric carcinomas (Fig. 6C-D and Supplementary Table 5). Given that these tumors were treated, the association observed between GSDMB/LC3B/Rab7 staining patterns and clinical behavior support the idea that GSDMB over-expression could promote protective autophagic response to therapy in HER2 positive cancer patients.

**Fig. 7:**
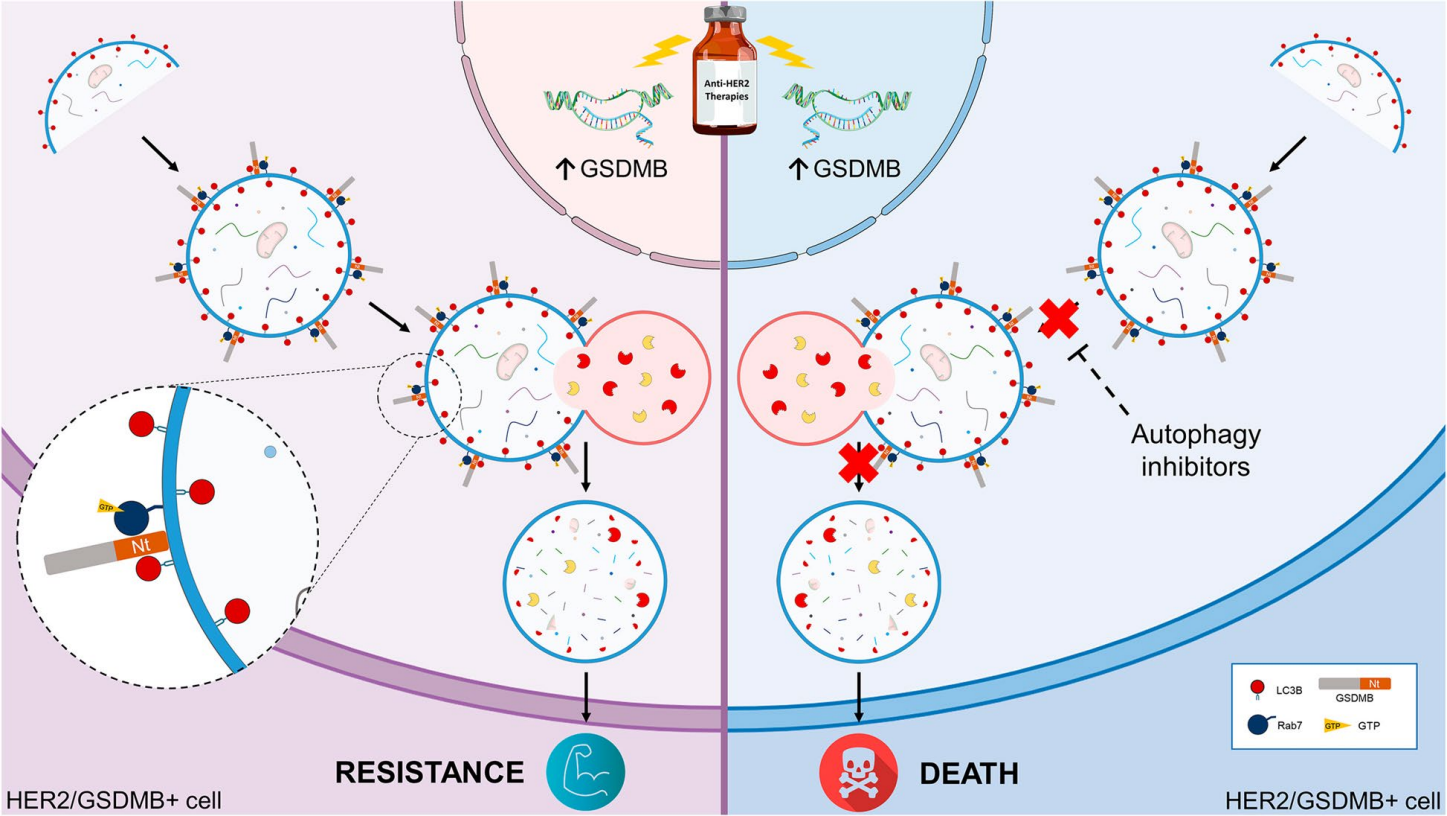
GSDMB acts as an autophagy adaptor in response to anti-HER2 therapies. Representative scheme of the proposed mechanism of action of GSDMB as mediator of protective autophagy response. It is highlighted the dualistic model on HER2/GSDMB over-expressing tumors behavior after the treatment anti-HER2 therapy (resistance phenotype) or its combination with autophagy inhibitors (sensible phenotype)

Taken together, our results reveal that the autophagy inhibition reverses, at least in part, the resistance to lapatinib mediated by GSDMB over-expression in HER2 cancer cells. Moreover, the multiprotein complex formed by GSDMB, Rab7 and LC3B plays a key role during the pro-survival autophagy process *in vitro* and *in vivo*. Therefore, the combination of autophagy inhibitors with anti-HER2 agents could provide new therapeutic options for HER2/GSDMB-overexpressing breast and gastroesophageal tumors (Fig. 7).

## Discussion

The multifunctional protein GSDMB plays complex roles in inflammatory pathologies and cancer. On the one hand, similar to other GSDM family members [2, 3], the released/activated GSDMB NT domain has pore-forming activity on biological membranes that can provoke lytic cell death of normal [5, 7], cancer cells [8] and intracellular bacteria [9]. On the other hand, GSDMB full-length protein (GSDMB-FL) exhibits additional functions in pathogenesis, including cell motility and epithelial barrier repair during inflammatory bowel disease [11], transcriptional control of disease-associated genes in asthma [10], promoting invasion and/or metastasis of cancer cells [13, 14, 45], metabolism regulation in tumors [45], and cancer therapy resistance [12, 14]. Since GSDMB is frequently over-expressed in diverse cancer types [3], where usually associates with poor prognosis [3], the activation of GSDMB pro-cell death function has been previously explored as a novel therapeutic approach using two different methods: a) an antibody-based nanomedicine that seemed to activate the protein cytotoxic activity in breast cancer xenografts *in vivo* [14]; b) triggering the proteolytic cleavage of GSDMB NT pore-forming domain by lymphocyte Granzyme-A [8] via pharmacological activation (anti-PD-1) of the antitumor immunity response [8]. Whereas both approaches yielded promising results, only partial tumor regression was achieved, and so far, it is still unproven if GSDMB-mediated pyroptosis actually occurs in clinical tumor samples. Therefore, to expand GSDMB potential as therapy target, in the present work, we aimed at identifying other druggable pathways, controlling GSDMB pro-tumor functions, that could be readily translated into the clinic. Specifically, we focused on tackling the mechanisms by which GSDMB mediates limited response to anti-HER2 therapy [12, 14] in tumors. While GSDMB over-expression in breast and likely gastric [44] cancer is mostly due to GSDMB-HER2 gene co-amplification, our results together with our published data [12], prove that GSDMB transcription and translation are further upregulated specifically in response (early response and during acquired resistance) to different anti-HER2 drugs (trastuzumab, lapatinib) in HER2 breast cancer (cell lines and PDXs) and gastroesophageal tumor cells. GSDMB can also be upregulated by different chemokines (IFN-α, -β, and -γ, or a lesser extent TNF–α) in cancer models [8], and methotrexate in gut epithelium [11], but the effects of this upregulation depends on the cellular and pathological settings. Thus, GSDMB can promote migration, adhesion and proliferation, through FAK phosphorylation changes mediated by PDGF-A, during gut epithelial barrier repair/restitution [11], while GSDMB effects on breast cancer invasion/metastasis and tumor growth depend on the cellular and *in vivo* contexts [12–14, 46]. Generally, GSDMB does not significantly affect breast tumor proliferation (as shown in this work and previous literature [12–14, 46]) but can mediate cancer survival upon therapy challenge. Here we prove that high GSDMB levels are required for anti-HER2 therapy resistance in breast and gastroesophageal cancers since GSDMB downregulation by si-/shRNAs or CRISPR/Cas9 significantly increases sensitivity and partially reverts the resistant phenotype in these tumors.

Importantly, we demonstrate for the first time that this GSDMB function requires the activation of protective autophagy. Autophagy, a catabolic mechanism where molecules and damaged cellular organelles are degraded, has a complex role in cancer development and clinical behavior, acting as either a pro-death or a pro-survival factor depending on the cancer type and stage as well as treatment [47]. In HER2 tumors, the anti-HER2 targeted drugs lapatinib [22] and trastuzumab [48] trigger protective autophagy to maintain the resistant phenotype [20], and accordingly, abrogation of autophagy by specific inhibitors (like chloroquine, CQ) can re-sensitize cancer cells to these drugs and enhance tumor cell death in preclinical models [20]. Nevertheless, the biological determinants affecting autophagic response to these drugs and the sensitivity to autophagy inhibitors are not well understood so far. Here, we discovered that GSDMB-high cells exhibit upregulated autophagic flux, and the inhibition of this process by CQ significantly enhances the response to lapatinib *in vitro*, inducing a shift from cell viability towards toxicity both in early response and in lapatinib-resistant cells. Interestingly, lapatinib plus CQ nearly abrogates tumor growth in two preclinical *in vivo* models (zebrafish, mouse), thus proving the therapeutic utility of this combination.

Mechanistically, our data indicate that GSDMB, Rab7 and LC3B form a complex after lapatinib and/or CQ treatment. The GSDMB-Rab7 functional axis is important for mediating survival autophagy, since Rab7a silencing increases the sensitivity to lapatinib of GSDMB-expressing and lapatinib resistant cells, and higher GSDMB levels upregulate Rab7 activation (measured by the co-immunoprecipitated levels of Rab7-GTP). Rab7 performs multiple functions that depend on the interaction with diverse downstream effectors [26]. In particular, Rab7 activity during autophagosome-lysosome fusion is regulated by its effector, FYCO1 (FYVE and coiled-coil domain-containing autophagy adaptor 1), which promotes microtubule plus end–directed transport of autophagic vesicles [49], and other partners, such as Nlp (ninein-like protein) which enhances autophagosome-lysosome fusion via promoting Rab7 and FYCO1 interaction [50]. Given that GSDMB is not an autophagic cargo (not degraded during autophagy), interacts with LC3B and induces Rab7 activation, we propose that GSDMB acts, like FYCO1, as an autophagy adaptor that specifically facilitates autophagosome maturation and the autolysosome formation in response to anti-HER2 therapies.

Further supporting this idea, we prove that a region of GSDMB NT domain (1–91 residues) harboring two LIR motifs is necessary for Rab7/LC3B interaction and the subsequent autophagy induction, since deletion of this region (construct GSDMB^92−416^) abrogates the co-immunoprecipitation and colocalization of Rab7/GSDMB and LC3B/GSDMB in HCC1954 GB cells. Moreover, similar to FYCO1, which function also depends on its binding to LC3 and phosphatidylinositol-3-phosphate (PI3P) [49], GSDMB NT domain has affinity to specific lipids such as mono-, bis- and tri-phosphoinositides and, weakly to sulfatides and cardiolipin [14, 30]. Thus, it is reasonable to speculate that GSDMB NT, at least the first 91 residues, might be important for the anchorage of GSDMB to autophagosomes, and its downstream effects on Rab7 activity, like other Rab7 regulators [26]. For instance, PI3P, essential in the initial steps of autophagy, is required by the Rab7 guanine nucleotide exchange factor (GEF), Mon1-Ccz1, for binding to early autophagosomes and the following activation of Rab7 [26].

It is worth noting that our results, together with previous studies based on other GSDMs, indicate that these proteins can regulate diverse autophagic processes that result in cell- and context-dependent biological consequences. For instance, GSDMA3 mutant proteins (with activated NT) provoke autophagy leading to cell death [4], whereas cleaved GSDMD NT localizes to autophagosomes in neutrophils, inducing autophagy-mediated cell-death-independent IL-1β secretion [41], and full length PJVK/DFNB59 recruits LC3-II to produce pexophagy, a type of selective autophagy for degrading damaged peroxisomes [42].

While further research is required to fully decipher the importance of the autophagy-related functions of GSDMs in disease conditions, our data in patient cohorts with HER2 gastric or breast carcinomas support that GSDMB/Rab7/LC3B-autophagy axis has an impact on clinical behavior. Indeed, treated gastric and breast cancer patients with higher puncta tumor expression of both GSDMB/Rab7 and GSDMB/LC3B are more prone to relapse. Our results are in line with previous studies describing GSDMB [12, 14], Rab7 [51] and LC3 over-expression [52, 53] as poor prognosis cancer factors and reinforce the connection between GSDMB/Rab7/LC3B co-expression, protective autophagy, resistance to anti-HER2 therapies and adverse prognosis in clinical samples.

Finally, and most notably, our findings have two important therapeutic implications for the future treatment of HER2/GSDMB tumors. First, they open a new therapeutic opportunity for lapatinib, whose use in current clinical practice is mostly restricted to the advanced stages in combination with chemotherapy and/or other HER2-directed targets [17]. Second, provide evidence that the FDA-approved chloroquine in combination with lapatinib can be a novel effective therapy for anti-HER2-drug resistant HER2/GSDMB positive tumors.

## Conclusions

Our study reveals for the first time that GSDMB acts like a pro-survival autophagy adaptor in response to anti-HER2 drugs, binding autophagosome surface-lipidated LC3B and promoting Rab7 activation. Moreover, we demonstrated the preclinical utility of combining the autophagy inhibitor CQ with the HER2-targeted drug lapatinib; thus, contributing to the evolving treatment paradigm in HER2 tumors overexpressing GSDMB, which are largely associated with poor outcomes.

## Supplementary Information

**Additional file 1.** **Supplementary Figure 1. **Upregulation of GSDMB in response to anti-HER2 therapies and expression of different GSDMs in GSDMB-silenced cells after treatment with lapatinib. **Supplementary Figure 2. **Lapatinib induces pro-survival autophagy in HCC1954 and OE19 cells. **Supplementary Figure 3. **GSDMB-high cells show an increased autophagic flux in response to starvation. **Supplementary Figure 4**. GSDMB-mediated autophagic response is not correlated with mitophagy, aggrephagy or lipophagy. **Supplementary Figure 5. **ATG5-silencing renders GSDMB-high cells more sensitive to lapatinib. **Supplementary Figure 6. **The combination of lapatinib plus chloroquine increases the therapeutic response *in vivo *in zebrafish xenografts of GSDMB-expressing tumors. **Supplementary Figure 7. **Immunohistochemical and histological analysis in orthotopic tumor xenografts. **Supplementary Figure 8. **GSDMB potentially forms a multiprotein complex with Rab7 and LC3B. **Supplementary Figure 9. **GSDMB knockout cells show a decreased autophagic flux, correlated with higher sensitivity to lapatinib treatment, compared to control cells. **Supplementary Table 1.** Immunohistochemical and clinical data of the HER2+ gastric carcinoma cohort and the HER2+ breast carcinoma series*. **Supplementary Table 2**. Relationship between GSDMB expression and clinical or immunohistochemical features in the adjuvant treated HER2-positive gastric carcinoma and breast carcinoma* cohorts. **Supplementary Table 3. In vivo **acute toxicity results in zebrafish. **Supplementary Table 4. **Summary of potential cancer and autophagy GSDMB interactors proteins obtained from immunoprecipitation and mass spectrometry. **Supplementary Table 5.** Co-expression of GSDMB and autophagy markers (LC3B and Rab7) and their associations with relapse in the adjuvant treated HER2-positive gastric carcinoma and breast carcinoma* cohorts. **Supplementary Table 6.** List of primary antibodies used for western blot (WB), immunofluorescence (IF), immunoprecipitation (IP), and immunohistochemistry (IHC).

## Data Availability

All data generated or analyzed during this study are included in this published article and its supplementary information files.

## Abbreviations

ATG5: Autophagy related 5
CQ: Chloroquine
CNS: Central nervous system
DMSO: Dimethyl sulfoxide
FAK: Focal Adhesion Kinase
FDA: U.S. Food and drug administration
FYCO1: FYVE and coiled-coil domain-containing autophagy adaptor 1
GSDM: Gasdermin
GSDMB: Gasdermin B
HER2: Human epidermal growth factor receptor 2
IF: Immunofluorescence
IHC: Immunohistochemistry
IP: Immunoprecipitation
Lap: Lapatinib
LC3B: Microtubule-associated proteins 1A/1B light chain 3B
LIR: LC3-interacting region
LR: Lapatinib resistant cells
NTC: Non-targeting control
PDGF-A: Platelet-Derived Growth Factor-A
PDX: Patients-derived xenografts
PI: Propidium iodide
Rab7: Ras-related protein Rab-7
RT-qPCR: Reverse transcription-quantitative polymerase chain reaction
TKI: Tyrosine kinase inhibitor
WB: Western blot
