# Supplementary material for: Gasdermin B over-expression modulates HER2-targeted therapy resistance by inducing protective autophagy through Rab7 activation
Source: J Exp Clin Cancer Res. 2022 Sep 26;41:285. doi: 10.1186/s13046-022-02497-w (PMC9511784; doi:10.1186/s13046-022-02497-w)
Supplement: Supplementary file 1 — Additional file 1. Supplementary Figure 1. Upregulation of GSDMB in response to anti-HER2 therapies and expression of different GSDMs in GSDMB-silenced cells after treatment with lapatinib. Supplementary Figure 2. Lapatinib induces pro-survival autophagy in HCC1954 and OE19 cells. Supplementary Figure 3. GSDMB-high cells show an increased autophagic flux in response to starvation. Supplementary Figure 4. GSDMB-mediated autophagic response is not correlated with mitophagy, aggrephagy or lipophagy. Supplementary Figure 5. ATG5-silencing renders GSDMB-high cells more sensitive to lapatinib. Supplementary Figure 6. The combination of lapatinib plus chloroquine increases the therapeutic response in vivo in zebrafish xenografts of GSDMB-expressing tumors. Supplementary Figure 7. Immunohistochemical and histological analysis in orthotopic tumor xenografts. Supplementary Figure 8. GSDMB potentially forms a multiprotein complex with Rab7 and LC3B. Supplementary Figure 9. GSDMB knockout cells show a decreased autophagic flux, correlated with higher sensitivity to lapatinib treatment, compared to control cells. Supplementary Table 1. Immunohistochemical and clinical data of the HER2+ gastric carcinoma cohort and the HER2+ breast carcinoma series*. Supplementary Table 2. Relationship between GSDMB expression and clinical or immunohistochemical features in the adjuvant treated HER2-positive gastric carcinoma and breast carcinoma* cohorts. Supplementary Table 3. In vivo acute toxicity results in zebrafish. Supplementary Table 4. Summary of potential cancer and autophagy GSDMB interactors proteins obtained from immunoprecipitation and mass spectrometry. Supplementary Table 5. Co-expression of GSDMB and autophagy markers (LC3B and Rab7) and their associations with relapse in the adjuvant treated HER2-positive gastric carcinoma and breast carcinoma* cohorts. Supplementary Table 6. List of primary antibodies used for western blot (WB), immunofluorescence (IF), immunoprecipitation (IP [file 13046_2022_2497_MOESM1_ESM.zip › GamezChiachio_et_al._Supplementary_Information_reviewedv3.docx]

**Supplementary Materials for**

**“Gasdermin B over-expression modulates HER2-targeted therapy resistance by inducing protective autophagy through Rab7 activation”**

Manuel Gámez-Chiachio^1,2#^, Ángela Molina-Crespo^1#^, Carmen Ramos-Nebot^1,2^, Jeannette Martinez-Val^3^, Lidia Martinez^1^, Katja Gassner^4^, Francisco J. Llobet^4^, Mario Soriano^5^, Alberto Hernandez^6^, Marco Cordani^1^, Cristina Bernadó-Morales^2,7,8^, Eva Diaz^9^, Alejandro Rojo-Sebastian^9^, Juan Carlos Triviño^10^, Laura Sanchez^4^, Ruth Rodríguez-Barrueco^11,^ Joaquín Arribas^2,7,121,13^, David Llobet-Navás^4^, David Sarrió^1,2^, Gema Moreno-Bueno^1,2,9^

1. Departamento de Bioquímica, Universidad Autónoma de Madrid (UAM), Instituto de Investigaciones Biomédicas ‘Alberto Sols’ (CSIC-UAM), IdiPAZ, Madrid, Spain.

2. Centro de Investigación Biomédica en Red de Cáncer (CIBERONC), Instituto de Salud Carlos III, Madrid, Spain.

3. Departamento de Zoología, Genética, Antropología Física, Universidad Santiago de Compostela, Lugo, Spain.

4. Mecanismos Moleculares y Terapia Experimental en Oncologia-Programa Oncobell, Idibell – L'Hospitalet de Llobregat, Spain.

5. Servicio de Microscopía Electrónica, Centro de Investigación Príncipe Felipe (CIPF), Valencia, Spain.

6. Servicio de Microscopía Óptica Avanzada, Centro de Investigación Príncipe Felipe (CIPF), Valencia, Spain.

7. Programa de Investigación Preclínica, Vall d'Hebron Institute of Oncology (VHIO), Barcelona, Spain.

8. Leitat Medical Department, Leitat Technological Center, Barcelona, Spain.

9. Fundación MD Anderson Internacional, Madrid, Spain.

10. Sistemas Genómicos, Paterna, Valencia, Spain.

11. Unidad de Anatomia, Departamento de Patologia y Terapèutica Experimental, Facultad de Medicina, Universidad de Barcelona (UB) – L'Hospitalet de Llobregat, Spain.

12. Programa de Investigación en Cáncer, IMIM (Hospital del Mar Medical Research Institute), Barcelona, Spain.

13. Institució Catalana de Recerca i Estudis Avançats (ICREA), Barcelona, Spain.

# Equal contribution

***Address correspondence to:** Gema Moreno-Bueno (gmoreno@iib.uam.es) & David Sarrió (dsarrio@iib.uam.es), Departamento de Bioquímica, UAM, Instituto de Investigaciones Biomédicas “Alberto Sols” (CSIC-UAM), C/Arturo Duperier 4, Madrid 28029, Spain

**This file includes :**

Supplementary Material and Methods

Supplementary Figures S1 to S9

Supplementary Tables S1, S2, S3, S5 and S6

**Supplementary Material and Methods**

**RNA interference, sgRNA design and overexpression constructs.** GSDMB-silenced HCC1954 and OE19 cells were generated using Mission® shRNA Lentiviral Transduction Particles (SHCLNV-NM_018530, Sigma-Aldrich, shGB1: TRCN0000137108 & shGB2: TRCN0000168794) according to the manufacturer’s instructions. Non-targeting Control shRNA Transduction Particles (SHC002V, Sigma-Aldrich) were employed as control (shNTC). To generate GSDMB knockout HCC1954 cells, we designed the sgRNAs (sgGB1: Forward, 5’-GTCGTCTCCCACCGAAGGGTAGCCAGATACTGCTGTTTCGAGACGTG-3’ and Reverse, 5’- CACGTCTCGAAACAGCAGTATCTGGCTACCCTTCGGTGGGAGACGAC-3’) by the tool CRISPRscan [1]. The resulting sgRNA was then cloned into the BsmBI sites of LentiCRISPR v2 plasmid (52961, Addgene). For HCC1954 LR and OE19 LR cells, GSDMB transient silencing was performed by two specific siRNAs (Custom siRNA, Dharmacon, siGB1: Sense sequence, 5'-GCCAAAGGGAAGUGACCAUUU-3’; Antisense sequence, 5'-AUGGUCACUUCCCUUUGGCUU-3’ & siGB2: Sense sequence, 5'-GAGAUUACCCAAAGAAAUAUU-3'; Antisense sequence: 5'- UAUUUCUUUGGGUAAUCUCUU-3'). ATG5 and Rab7 were silenced by a siGENOME Human ATG5 siRNA – SMARTpool (M-004374-04-0010, Dharmacon) and by a siGENOME Human Rab7a – SMARTpool (M-010388-00-0010), respectively. siGENOME non-targeting siRNA (D-001210-01-20, Dharmacon) was utilized as control (siNTC). Transfections were carried out using DharmaFECT 1 Transfection Reagent (T-2001-02, Dharmacon) according to the manufacturer's protocol. HCC1954 cells overexpressing myc-tagged full-length GSDMB (GB) [2] and a GSDMB construct lacking the residues 1-91(GB^92-416^) were obtained by stable lentiviral infection using the pLVX vector. The coding region of GB^92-416^, tagged with a myc-epitope in the C-terminal domain (highlighted in bold), was amplified by PCR using the primers: Forward, 5’-GCGAATTCATGTCTACGGGCGAGCTGAT-3’; Reverse, 5’-GCCCCACTAGCGTGAGCAGC**GAACAAAAACTCATCTCAGAAGAGGATCTG**TAGTCTAGA-3’**.** The resulting GB^92-416^-myc cDNA was then cloned into the EcoRI and XbaI sites of the pLVX-Puro plasmid (125839, Addgene). Lentiviral particles were generated in HEK293T by transfection (Lipofectamine™ 2000 Transfection Reagent, Invitrogen) with PAX2, VSVG and pLVX-Puro-GB (92-416) plasmids. For all the constructs, stably infected cells were selected using 0.5 µg/ml puromycin.

**Western blot and Co-immunoprecipitation.** Protein extracts from cultured cells were obtained using a standard 2% SDS buffer with protease and phosphatase inhibitors for 30 min at 4ºC. Equal amount of proteins measured using the Pierce™ BCA Protein Assay Kit (Thermo Scientific) were resolved by SDS-PAGE and analyzed by immunoblot. Membranes were incubated with the antibodies indicated in Supplementary Table S6. Amersham ECL HRP-conjugated anti-mouse or anti-rabbit secondary antibodies were used (NA931 and NA934, GE Healthcare Life Sciences, 1:5000 dilution). Detection was carried out with Pierce™ ECL Western Blotting Substrate (Thermo Scientific) and quantification was carried out by densitometric scanning and normalized to GAPDH expression. For co-immunoprecipitation, 2x10^6^ HCC1954 cells were lysed in a buffer containing 50 mM Tris-HCl (pH 7.5), 137 mM NaCl, 1% Glycerol, 1% Triton™ X-100in presence of a mixture of proteases inhibitors (Complete™, Mini Protease Inhibitor Cocktail, Roche) [3]. 2-4 mg of protein lysate was incubated overnight at 4 ºC with the Rab7 (9367, Cell Signaling Technology) or LC3B (sc-376404, Santa Cruz Biotechnology) antibody followed by incubation with Dynabeads™ protein G (Invitrogen) for 3 h at 4°C. After washing the beads with the lysis buffer three times, the beads-protein binding was detected by western blotting.

**Immunofluorescence staining.** Cells were grown to 50-70% confluency on coverslips in 24-well plates. After treatment, cells were fixed with 4% paraformaldehyde for 20 min at room temperature (RT). After 5 min permeabilization with 0.1% Triton™ X-100, coverslips were incubated with corresponding primary antibodies (Supplementary Table S6), followed by incubation with secondary antibodies, Alexa Fluor 488 or 546-labeled (A-11029, A-11034, A-21131 and A-11035, A-21127 Invitrogen) for 1 h at RT. Cell nuclei were stained with 1:5000 DAPI (D1306, Invitrogen). Images were captured on a LSM710 confocal microscope (ZEISS), analyzed, and quantified using ImageJ software. Colocalization analyses were performed by calculating the Manders' Overlap Coefficient, using the JACoP plugin on ImageJ [4].

**Drugs and autophagy analysis.** Trastuzumab (Herceptin, Roche) was directly diluted on cell culture medium with inactivated FBS and used at final concentrations of 1 mg/ml (HCC1954 cells) or 400 μg/ml (OE19 and NCI-N87). Lapatinib (Tykerb, GlaxoSmithKline) was dissolved in DMSO (Merck) to a stock concentration of 2.5 mM and used at 2µM (HCC1954), 0.7µM (OE19) or 0.15 µM (NCI-N87). Chloroquine diphosphate salt (CQ, Sigma-Aldrich) was applied at 10µM (HCC1954) or 50 µM (OE19). The autophagic flux quantification was performed measuring LC3B-II expression by WB as described before [5,6]. Briefly, two different relative LC3B-II expression measures were obtained from each cell line: basal condition and autophagy induction after lapatinib treatment. Basal autophagy quantification was obtained by subtracting the measure of LC3B-II of untreated cells from the LC3B-II of CQ treated cells (both measures normalized to GAPDH). On the other hand, the induced autophagy quantification was obtained similarly by deducting the measure of LC3B-II of lapatinib treated cells from the LC3B-II of cells treated with the combined regimen. For that purpose, 15 × 10^4^ HCC1954 and 60 × 10^4^ OE19 cells/well were seeded in 6-well plates. After 24 h, cells were treated for 72 h with CQ and/or lapatinib or starvation (complete media with 0.01% FBS) with/without CQ. For the analysis of LC3B by immunofluorescence, HCC1954 and OE19 cells were grown on coverslips to 50-70% confluency and then treated for 24 h with IC50 of lapatinib and/or CQ.

**Cell viability assay.** The cytotoxic effect of analyzed compounds on HCC1954, OE19 and NCI-N87 was tested using the AlamarBlue reagent (Bio-Rad). Briefly, 5x10^3^ HCC1954, 2x10^4^ OE19 and NCI-N87 cells/well were seeded into a 96-well plate in triplicates. After 24 h, cells were treated with previously indicated concentrations of specific treatments, alone or in combination, for 72 h. Spectrophotometric analysis was performed at wavelengths of 570 and 600 nm.

**Cell apoptosis analysis.** Apoptotic rate was quantified using the Annexin V-FITC stained apoptosis detection kit (Immunostep) and propidium iodide (PI, Sigma-Aldrich) according to the manufacturers’ instructions. HCC1954 and OE19 cells were treated with lapatinib and/or CQ at previously described concentrations for 72 h. Annexin V-FITC positive cells alone (A+/PI-) and Annexin V-FITC and PI doubled stained (A+/PI+) were defined as apoptotic cells. The apoptosis analysis was conducted using a FC500 flow cytometer (Beckman Coulter).

**Transmission Electron Microscopy.** HCC1954 cells treated with different regimens were fixed in 3% glutaraldehyde in 0.1 M cacodylate buffer for 2h. After several washes with 0.1 M cacodylate buffer, post-fixation was carried out with 1% osmium tetroxide and 0.8% aqueous potassium ferrocyanide. Then, fixed cells were dehydrated and embedded in Durcupan ACM epoxy resin (Sigma-Aldrich). Ultrathin sections (60 nm) were obtained with a Leica Reichert Ultracut S Ultramicrotome (Leica Biosystems) and were then stained with 2% aqueous uranyl acetate and lead citrate (Reynolds). Images were captured on a JEM 1010 transmission electron microscope (JEOL), equipped with a ORIUS SC200 camera (Gatan). Analysis of the relative volume density of autophagic vacuoles was performed as previously described [7], using the Grid tool on ImageJ.

**RNA isolation and RT-qPCR.** Total RNA was extracted from cells with the TRIzol™ Reagent (Invitrogen) and the RNeasy Mini Kit (Qiagen) following standard procedures. Total RNA from the primary cultures derived from HER2+ PDX in which lapatinib and trastuzumab resistance were induced as previously described [8] was isolated using the ReliaPrep™ RNA Miniprep System (Promega), according to the manufacturer’s protocol. DNase treatment was performed to avoid the presence of residual DNA. cDNA reverse transcription was carried out with the M-MLV RT (VWR Chemicals). GSDMB isoforms qPCR was conducted using TaqMan gene expression assay primers (GSDMB-1: Hs00938445_m1; GSDMB-2: Hs00939390_m1; GSDMB-3&4: Hs00940508_m1; GSDMB-4: Hs00940509_m1; and GAPDH: Hs02758991_g1; Thermo Fisher Scientific). Each PCR reaction was performed in triplicate using the PerfeCTa™ qPCR FastMix UNG ROX (Quantabio). Expression values for each gene were normalized to housekeeping gene GAPDH expression. The analysis of HER2 and GSDM genes was performed in triplicate using the PerfeCTa™ SYBR^®^ Green FastMix™ (Quantabio) and the following primers:

GSDMA (F: 5’-AAACCTTCCCTCCTGGAGAA-3’, R: 5’-CCCTTGTCTA GAGCCCCTTC-3’),

GSDMB (F: 5’-TGGCTACCCTTGAAAACAGG-3’, R: 5’-ATATTGCCGGTCGCTTTTC-3’), GSDMC (F: 5’-AGCTCAGGTCATTTGGATGG-3’, R: 5’-GCACCATTATGGCTTC AAGG-3’), GSDMD (F: 5’-GCGTCAGGTTGCAGTTTCAC-3’, R: 5’-CGGACTACCCGCTCAAAGG-3’), GSDME (F: 5’-GAAGATCA CGACGATGCAGA-3’, R: 5’-GAAGGCAGAACTCGAACTGG-3’), HER2 (F: 5’-CGAGTGTCAGC CTCAAAACA-3’, R: 5’-TCATCCAGATCCACACAGGA-3’), GAPDH (F: 5’-TGCCATCAATGACCCCTTC-3’, R: 5’-ATTTGCCATGGGTGGAATC-3’). All RT-qPCR experiments were performed and analyzed in a StepOnePlus™ Real-Time PCR system (Applied Biosystems).

**Zebrafish studies.** Wild-type zebrafish (AB strain, *Danio rerio*) were maintained in a controlled aquatic facility at 27ºC (± 1ºC) and 14/10 h light/dark light cycle, according to previously described recommendations [9]. First, to determine several toxicological parameters of lapatinib and CQ in zebrafish model, the Fish Embryo Acute Toxicity test (FET, following the recommendation of the Organization for Economic Co-Operation and Development (OECD) No. 236, 2013) was performed. Briefly, 72 hours post-fertilization (hpf) hatched embryos were incubated in 24-well plates with different concentrations of the tested drugs for 96 h. Osmosis water and 0.1% DMSO (Merck) were used as negative controls. Three replicates were performed, analyzing a total amount of 60 embryos per concentration. Treated embryos were checked under an inverted optical microscope (Nikon TMS) at 24, 48, 72 and 96 h, in accordance with the guideline test and following the FET test scheme described previously [10]. ToxRat software was used to determine the 10% lethal concentration (LC10), 50% lethal concentration (LC50), lowest observed effect concentration (LOEC) and non-observable effect concentration (NOEC) values of the tested drugs. The LC50 values (35.1 µM and 116.4 µM for lapatinib and CQ, respectively) were higher than *in vitro* approaches (2 μM, lapatinb and 10 mM for CQ) confirming that drug concentrations used for xenograft treatment were appropriate as they were not harmful to the host embryos. Next, to analyze the *in vivo* efficacy of the lapatinib and/or CQ, 1x10^6^ of HCC1954 GSDMB expressing and silencing cells (shGB1/shGB2) as well as HCC1954 LR and C resistant cells (expressing green fluorescent protein, GFP) were inoculated into the perivitelline space of fish embryos of 48 hpf (n= 40-50 embryos per condition). Previously to injection, embryos were anesthetized with 0.003% tricaine (Sigma-Aldrich) and GFP transduced cells were resuspended in 10 μl 2% Polyvinylpyrrolidone (Sigma-Aldrich). Injected embryos were incubated at 34ºC until the end of the experiments. Xenografts were photographed at 24 hpi (hours post-injection) and before drug treatment (0 hpt) with an AZ-100 fluorescence stereomicroscope (Nikon). Then, embryos were randomly distributed in the treatment groups: control (0.1 % DMSO, vehicle), lapatinib (2 µM) and a combination of lapatinib with CQ (10 µM). After 48 hpt, xenografts were photographed again to analyze the tumor growth. At the end of experiments, embryos were euthanized by tricaine overdose. Three experiments were performed obtaining at least 60 embryos per each condition. Tumor growth and treatment response were analyzed measuring the fluorescence intensity ratio (48 hpt/0 hpt) using an image analysis program (QuantiFish [11]). These ratio values were: 1, when tumor size was maintained; >1, when tumor cells proliferated during the incubation time with the treatment and <1, when tumor cells died after 48 h of incubation (possible outliers, analyzed with the outlier calculator tool of GraphPad software, were discarded). *In vivo* zebrafish acute toxicity parameters were obtained using the ToxRat software. Lethal concentrations (LC) were determined using a linear maximum likelihood regression from the 96 h survival data and with 95% confidence limits. NOEC and LOEC values were obtained using a qualitative trend analysis by contrast, step-down Cochran-Armitage test and Tarone’s test. In accordance with OECD guideline, tests were considered valid whether the zebrafish embryos mortality in the negative control was less than 10% and the mortality in the positive control was more than 30%. The number of samples (n) has been indicated for each figure panel. In all cases, *P* values < 0.05 were considered as statistically significant.

**Mouse studies.** Orthotropic breast tumor xenografts were performed in female nu/nu mice (Charles River) following standard procedures^2^. mCherry-luc transduced shNTC, shGB1 or shGB2 HCC1954 (1x10^6^) cells were injected into the right fifth mammary fat pad (mfp) of 7-week-old female NMRI-nu Immunodeficient Mice (Janvier Labs) and tumor growth was assessed as described previously [2,12]. When tumor diameter reached approximately 0.5 cm, the animals were randomized into four groups of treatment, with at least five animals in each group: control (vehicle-treated), lapatinib alone, CQ and the combination of lapatinib + CQ. Lapatinib was resuspended in an aqueous solution containing 0.1% Tween 80 (Sigma-Aldrich) and 0.5% Hypromellose (Sigma-Aldrich) and delivered orally at 100 mg/kg once daily [13]. CQ was solubilized in sterile 1X phosphate-buffered saline (PBS) and administered intraperitoneally at 50 mg/kg once daily [14,15]. Mice were weighed and tumor growth was monitored twice a week. These experiments were done following the humane endpoint criteria for early termination for oncologic studies specified by Orellana-Muriana et al [16]. Briefly, these criteria include tumor larger than 1 cm in untreated animals, presence of tumor ulceration or body weight loss >20%. Furthermore, the signs of pain or distress were measured according to N3CRs mice grimace scale [17] and body condition scores [18]. Tumor size was measured using a caliper and the tumor volume was calculated using the formula L x W^2^ x (π/6), where L is the length and W, the width of the tumor xenografts. At the end of the experiment, the mice were sacrificed, and the tumors extracted and processed for histological evaluation. To assess cancer mass, excised tumors were pierced with scissors to remove liquid and then tumor weight was measured with a precision scale. A hematoxylin and eosin (H&E) staining and GSDMB and PCNA immunohistochemical analysis was performed in all tumors as described below.

**Immunohistochemistry analysis.** Human and mouse tumor samples were collected, fixed in 4% formaldehyde, and embedded in paraffin. Three µm thick sections were stained with H&E solutions. Briefly, tumor sections were deparaffinized using the BOND Dewax Solution (Leica Biosystems) and rehydrated with serial passage through changes of decreasing ethanol solutions. Immunostaining was performed on a BOND RX^m^ autostainer (Leica Biosystems) using BOND Epitope Retrieval Solutions (Leica Biosystems) and the BOND Polymer Refine Detection kit (Leica Biosystems), following the manufacturer’s instructions. Stained slides were counterstained with hematoxylin and coverslipped for obtaining the images with an Aperio CS2 scanner (Leica Biosystems) that were analyzed with the Aperio eSlide Manager software (Leica Biosystems). Immunohistochemical analyses in tumor sections of GSDMB, LC3B and Rab7 were performed according to stablished protocols [5,19]. A semiquantitative scoring of each marker based on staining intensity and the percentage of stained tumor cells was applied by two independent observers, blinded to clinical data.

**Multiplex immunofluorescence staining.** Human tumor samples were fixed in 4% formaldehyde and embedded in paraffin. Two µm thick sections were deparaffinized during 30 min at 65 ºC. Immunostaining was performed on a BOND RX^m^ autostainer (Leica Biosystems) using the Opal TM 7-color Automation IHC Kit (NEL82100T, Akoya Biosciences), with Opal-520, Opal-650, and Opal-570 used to detect GSDMB, LC3B and Rab7, respectively, following the manufacturer´s instructions. Images were captured on a LSM710 confocal microscope (ZEISS). Primary antibodies used are listed in Supplementary Table S6.

**Proteomic study.** GSDMB proteomic analysis was done at the PROTEOMICA-Unit-UCM following the Unit standard protocols. Briefly, after immunoprecipitation assays of HCC1954 C (pLVX empty plasmid), GB, GB^(92-416)^ using a Myc-tag antibody, the immunoprecipitated proteins were resolved on a 4% SDS-PAGE gel and stained with Coomassie Brilliant Blue G-250 (20279, Thermo Scientific). Then, trypsin digestion was placed. Isolated proteins were reduced with 10 mM DTT at 56ºC during 1 h and alkylated with Iodoacetamide 55 mM during 30 min on dark following by 0,8 ug trypsin digestion (Roche Molecular Biochemicals) at 37 ºC during 12 h. Obtained peptides were dried using a Savant SpeedVac Vacuum concentrator (Thermo Fisher Scientific) and reconstituted with 2X acetonitrile and 0.1% formic acid. Peptides were identified using liquid nano-chromatography (nano Easy-nLC 1000, Thermo Fisher Scientific) associated with high resolution mass spectrometry Q-Exactive HF (Thermo Fisher Scientific). Peptide concentration was performed “on-line” reverse phase chromatography (RP) using Acclaim PepMap 100 column (164569, Thermo Scientific) and then, separated by analytic reverse phase column C18 PicoFrit (NTCC-360/75-3-125, Nikkyo Technos Co.,Ltd). Peptide identification was perfomed with 60.000 resolution on Full scan (m/z 350-2000 Da). MS/MS acquisition data was obtained using Data-dependent acquisition (DDA). MS/MS acquired spectrums was done using the Proteome Discoverer 2.2 (Thermo Scientific) software using Human Swissprot data (42252 sequences on 20/02/2018). Unspecific proteins identified in the proteomic analysis of HCC1954 C cells were subtracted from the subsequent data analyses of HCC1954 GB and GB^(92-416)^ conditions .

**Rab7 activation assay.** After lapatinib alone or lapatinib plus chloroquine treatment, cells were collected to measure Rab7GTP using the Rab7 Activation Assay Kit (82501; NewEast Biosciences) according to the manufacturer’s instructions.

**Aggresome formation analysis by flow cytometry**. Aggresome formation was determined using the Aggresome Detection Kit (Abcam, ab139486) according to the manufacturer’s instructions. Briefly, 500.000 cells were seeded per well of a 6-well cell culture plate and were treated with 2 μM lapatinib alone or in combination with 10 μM chloroquine. After respective treatments, cells were washed with PBS, trypsinized, fixed with 4% paraformaldehyde and permeabilized (0.5% Triton X-100, 3 mM EDTA, pH 8). After additional washes with PBS, cells were incubated with 1:100000 Aggresome Detection Reagent diluted in 1x Assay Buffer for 30 min at room temperature. Aggresome staining was detected by flow cytometry (Gallios1, Beckman Coulter). Data was analysed using the Kaluza Analysis Software (Beckman Coulter). Using the geometric mean fluorescence intensity (MFI), the aggresome propensity factor (APF) was calculated as follows, APF = 100 x (MFI treated-MFI control) / MFI treated.

**In silico Protein Prediction tools.** iLIR database (https://ilir.warwick.ac.uk/) was used to detect LIR motifs on the full-length GSDMB protein [20]. The in silico study of GSDMB, LC3B and Rab7a interaction were predicted using the swiss-model algorithm [21]. The GSDMB, LC3B and Rab7a 3D structures were obtained from Uniprot data bases (accession number Q8TAX9, Q9GZQ8 and P51149, respectively). Then, the GSDMB and Rab7a protein-protein complex was deduced using InterEvDock2 [22] and PPcheck [23] webservers which allow to predict the interacting residues and the energetic stability estimation, respectively. Furthermore, the multiprotein complex GSDMB-Rab7a-LC3B was tested using the HawkDock webserver [24]. This method allows selecting the possible protein complex according to the predicted Gibbs energy. The structure with the least Gibbs energy, in this case, -2 kJ/mol, was selected.

**Supplementary Figures**

**
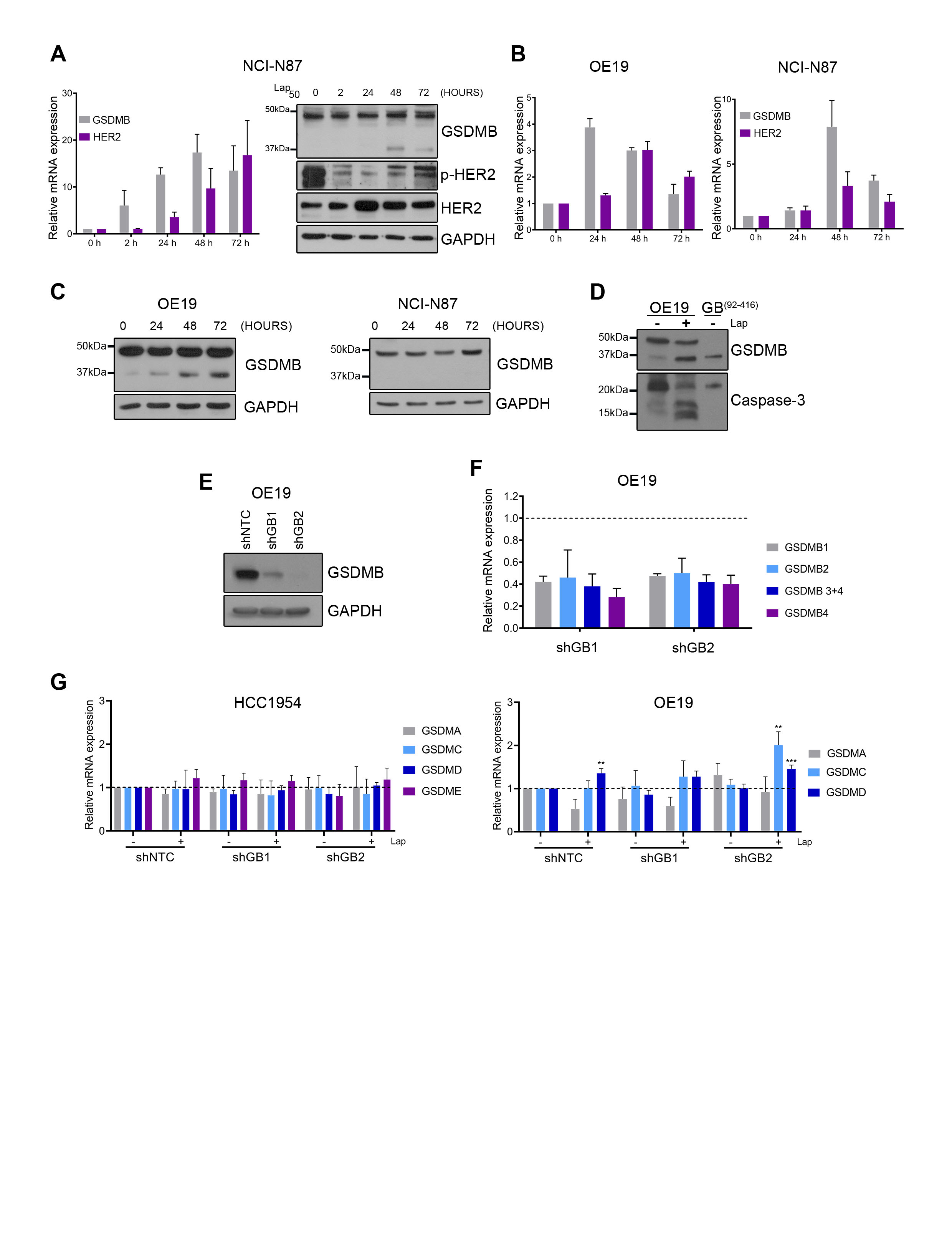
**

**Supplementary Figure 1.** Upregulation of GSDMB in response to anti-HER2 therapies and expression of different GSDMs in GSDMB-silenced cells after treatment with lapatinib. **A:** Relative mRNA (left) and protein levels (right) of GSDMB and HER2 in NCI-N87 cells treated with IC50 of lapatinib (0.15 µM) at indicated time points. **B-C:** Relative mRNA (**B**) and protein levels (**C**) of GSDMB and HER2 in OE19 (left) and NCI-N87 (right) cells treated with IC50 of trastuzumab (400 μg/ml in both cases) at different time points. **D:** GSDMB and caspase-3 analyses on OE19 control, treated with lapatinib and over-expressing GB^92-416^ cells. GB^92-416^ is the C-terminal fragment previously described [25] and generated after the caspase 3 processing. **E-F:** GSDMB protein (the four translated GSDMB isoforms, **E**) and mRNA (**F**) levels were stably decreased in OE19 cells by two specific shRNAs (shGB1 and shGB2), in comparison with the control (shNTC; dotted line). **G:** GSDMB-silencing effect in the mRNA levels of the other Gasdermin genes (GSDMA, GSDMC, GSDMD and GSDME) in HCC1954 (left) and OE19 (right) cells treated with or without lapatinib (2 µM and 0.7 µM, respectively, for 72 h). Note that OE19 cells exhibited no detectable expression of GSDME. Statistical significance was determined by two-tailed unpaired *t*-test (**P* < 0.05; ***P* < 0.01; ****P* < 0.001). Gene expression was normalized to GAPDH levels. Data are shown as the mean ± s.e.m. Three independent experiments with similar results were performed. Lap, lapatinib.


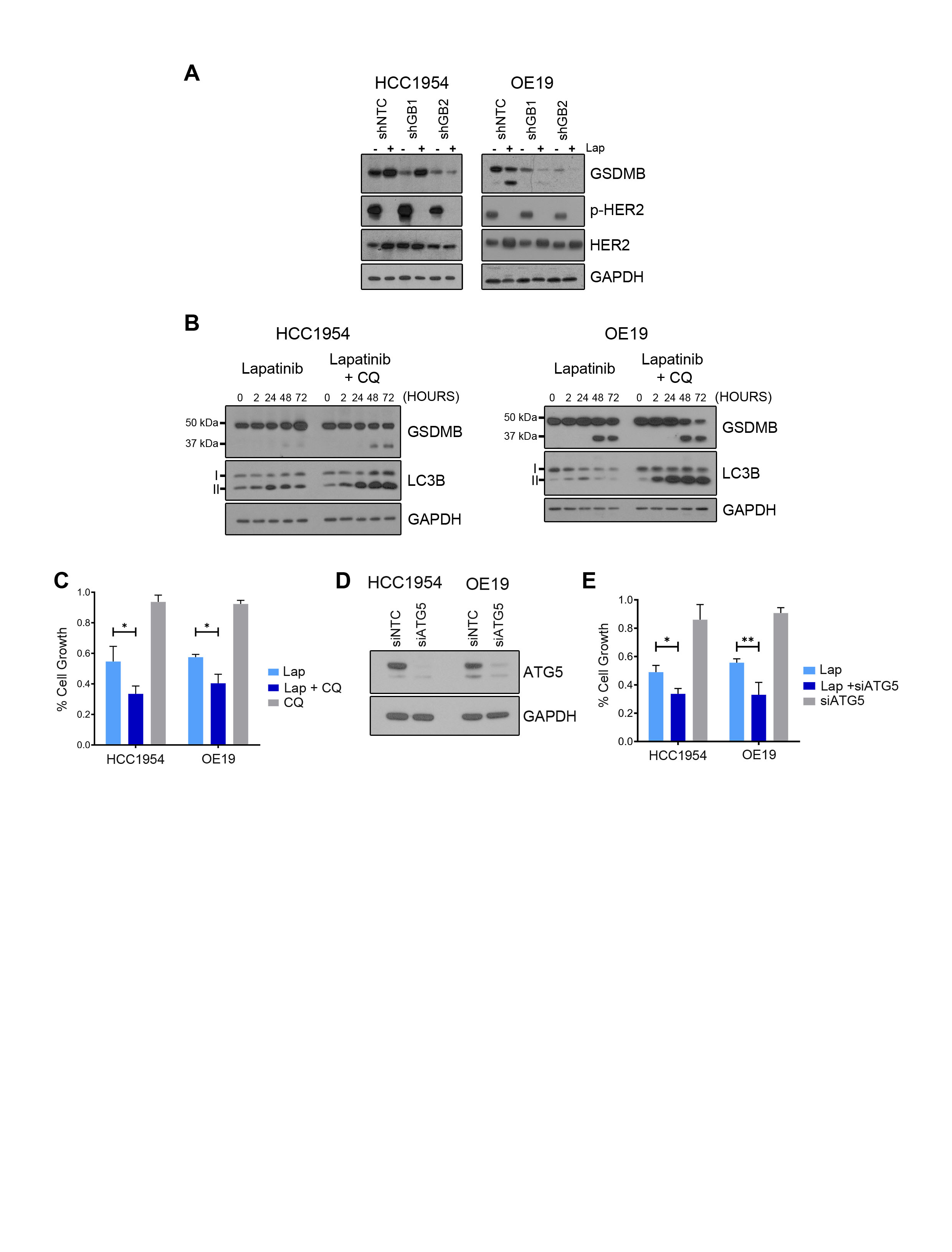


**Supplementary Figure 2.** Lapatinib induces pro-survival autophagy in HCC1954 and OE19 cells. **A:** GSDMB-silencing effect on the levels of HER2 receptor in HCC1954 and OE19 cells treated with or without IC50 of lapatinib (2 µM and 0.7 µM, respectively, for 72 h). **B:** GSDMB and LC3B protein levels in HCC1954 (left) and OE19 (right) cells treated with lapatinib (2 µM and 0.7 µM, respectively) or lapatinib + CQ (10 µM and 50 µM, respectively) at different time points. **C:** The cytotoxic effect of the treatment with lapatinib and/or CQ in HCC1954 and OE19 cells was evaluated by cell viability assays. **D:** ATG5 expression was effectively decreased in HCC1954 and OE19 cells by a specific siRNA (siATG5), compared to the control (siNTC). **E:** Similarly, to CQ treatment, the autophagy inhibition by siATG5 improves the effect of lapatinib in HCC1954 and OE19 cells, measured by cell viability assays. Three independent experiments with similar results were carried out. In (**C** and **E**), statistical significance was determined by two-tailed unpaired *t*-test (**P* < 0.05; ***P* < 0.01). CQ, chloroquine, NTC, non-targeting control. Lap, lapatinib. Data are shown as the mean ± s.e.m.


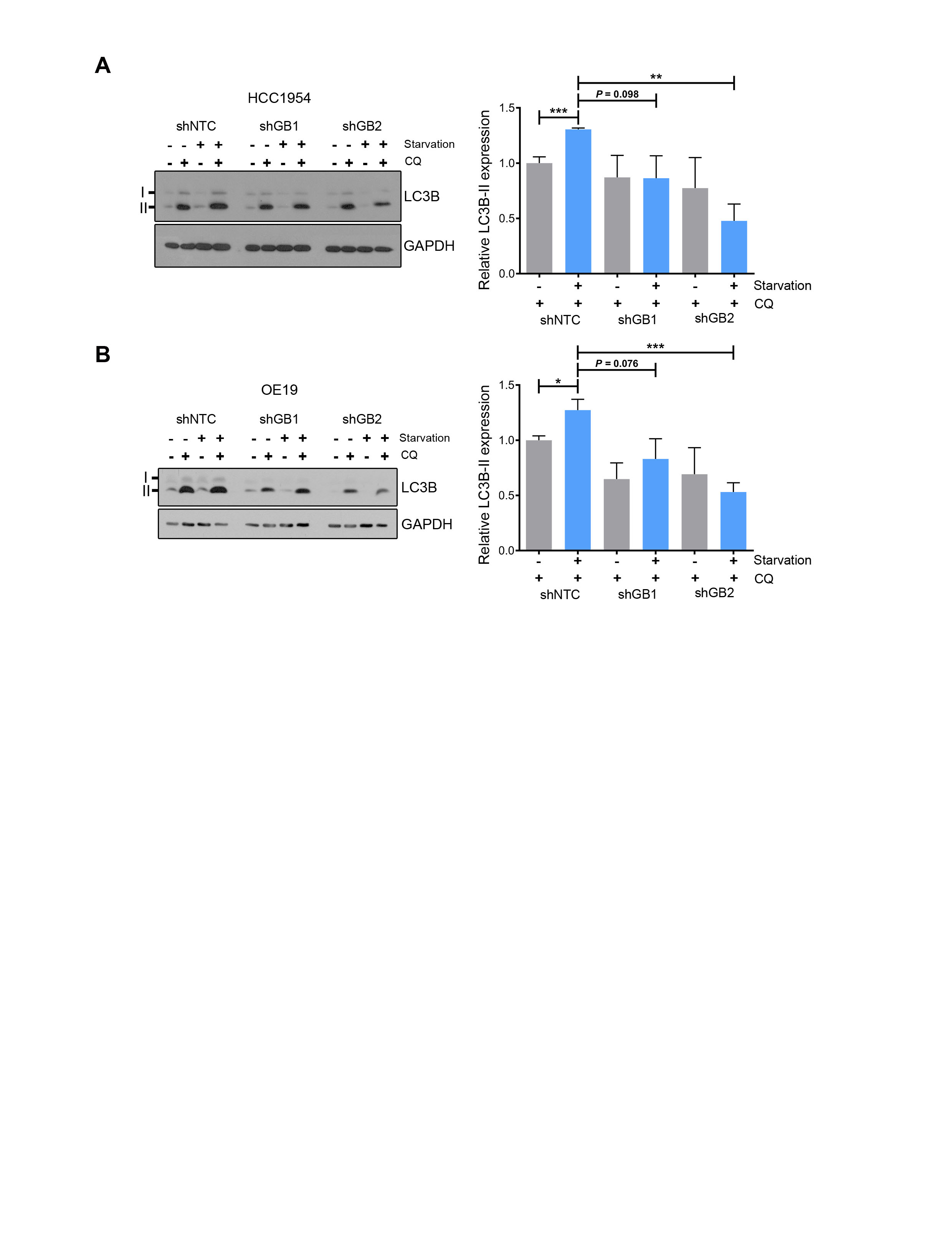


**Supplementary Figure 3.** GSDMB-high cells show an increased autophagic flux in response to starvation. **A-B:** GSDMB and LC3B protein levels in shNTC, shGB1 and shGB2 HCC1954 (**A**) and OE19 (**B**) cells treated with CQ (10 µM and 50 µM, respectively) and/or serum starved (0.01% fetal bovine serum) for 72 h. Quantification of LC3B-II expression was carried out by densitometric scanning and normalized to GAPDH expression Statistical significance was determined by two-tailed unpaired t-test (**P* < 0.05; ***P* < 0.01; ****P* < 0.001; ns, nonsignificant). Data are shown as the mean ± s.e.m. Three independent experiments with similar results were carried out. CQ, chloroquine, NTC, non-targeting control.


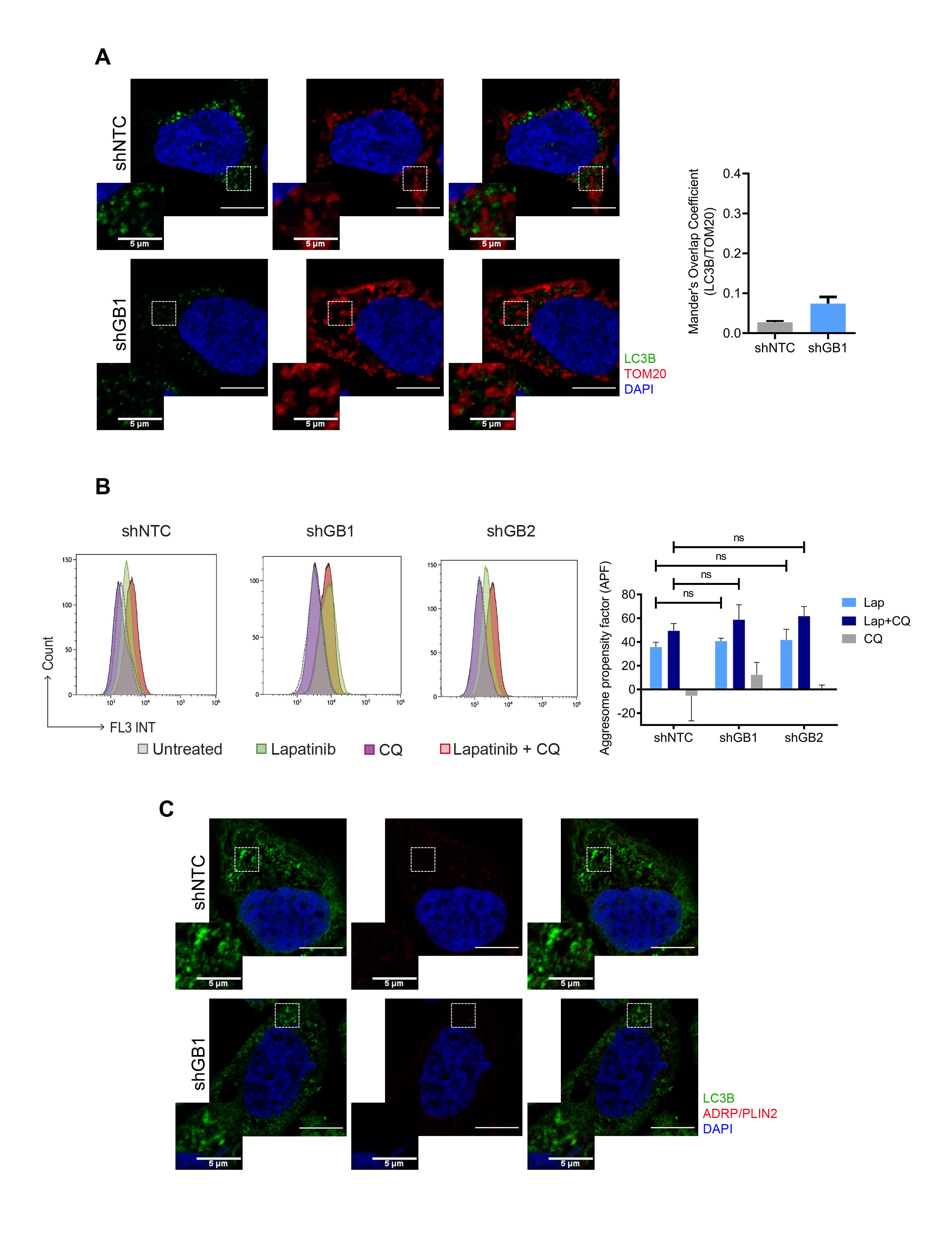


**Supplementary Figure 4**. GSDMB-mediated autophagic response is not correlated with mitophagy, aggrephagy or lipophagy. **A:** Representative images of the colocalization between LC3B (green) and the mitochondrial marker, TOM20 (red) by confocal microscopy in shNTC and shGB1 HCC1954 cells after lapatinib (2 µM) plus CQ (10 µM) treatment. Nuclei were counterstained with DAPI. Quantification of the Manders’ Overlap Coefficient (LC3B overlapping TOM20) is shown on the right. Manders’ Overlap Coefficients between 0 and 0.2 are not considered as positive colocalization. Three independent experiments were performed obtaining at least 15 cells, per experimental condition. Scale bar, 10 µm. **B:** Representative images of Aggresome Detection Reagent staining detected by flow cytometry (left) and quantification of the Aggresome propensity factor (APF, right) in shNTC, shGB1 and shGB2 HCC1954 cells after the treatment with lapatinib (2 µM) and/or chloroquine (10 µM) for 72 h. Three independent experiments with similar results were carried out. Statistical significance was determined by two-tailed unpaired t-test (ns, nonsignificant). **C:** Representative images of the no colocalization between LC3B (green) and the marker of the lipid droplets, ADRP/Perilipin 2 (red) by confocal microscopy in shNTC and shGB1 HCC1954 cells after lapatinib (2 µM) plus CQ (10 µM) treatment. Nuclei were counterstained with DAPI. Scale bar, 10 µm. Lap, lapatinib, CQ, chloroquine.


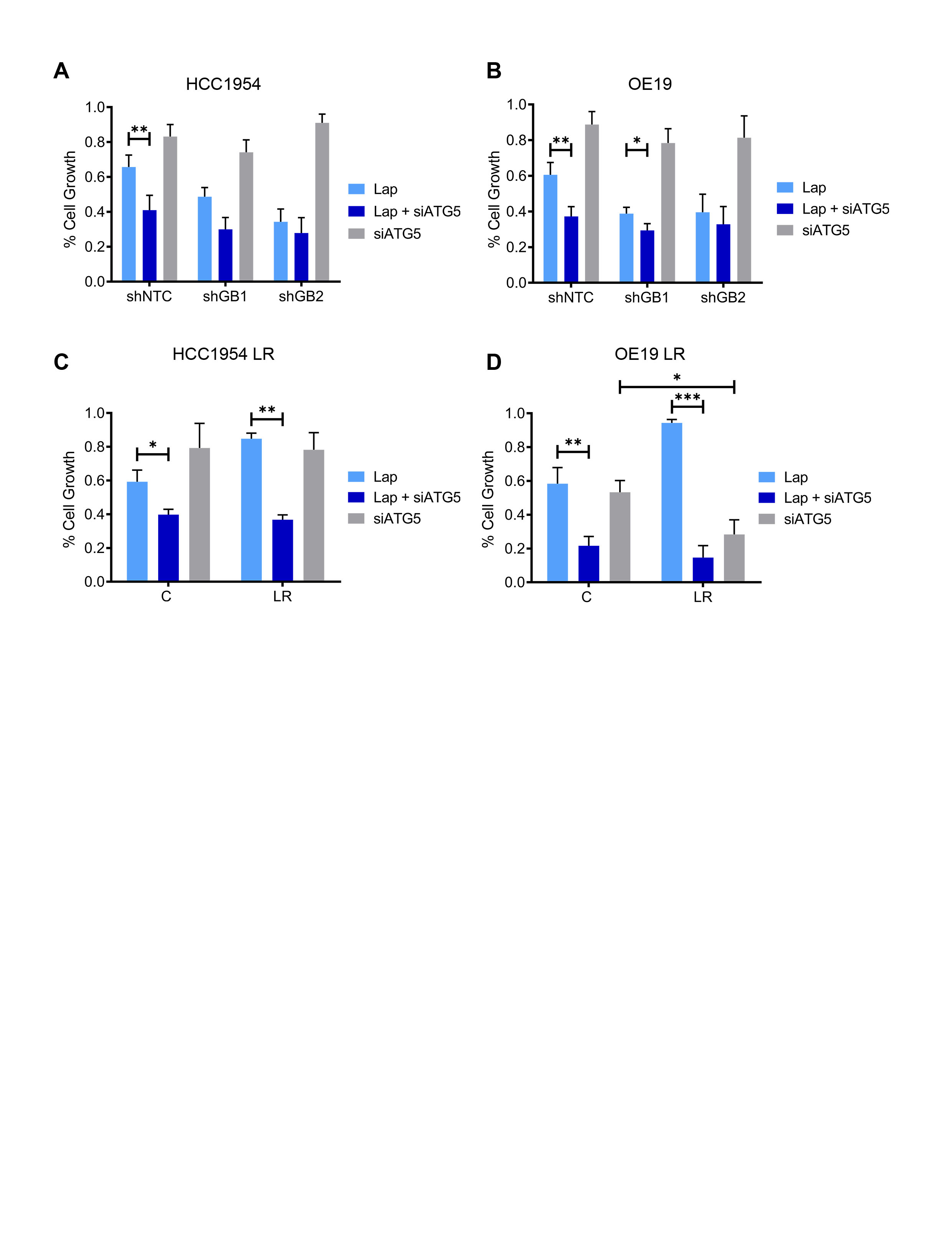


**Supplementary Figure 5.** ATG5-silencing renders GSDMB-high cells more sensitive to lapatinib. **A-D:** The cytotoxic effect of the treatment with lapatinib plus/or ATG5-silencing was evaluated by cell viability assays in shNTC, shGB1 and shGB2 HCC1954 (**A**) and OE19 cells (**B**), as well as HCC1954 LR (**C**) and OE19 LR cells (**D**). Statistical significance was determined by two-tailed unpaired t-test (*P < 0.05; **P < 0.01; ***P < 0.001). Data are shown as the mean ± s.e.m. Three independent experiments with similar results were performed. NTC, non-targeting control. LR: Lapatinib resistant cells. Lap, lapatinib.

**
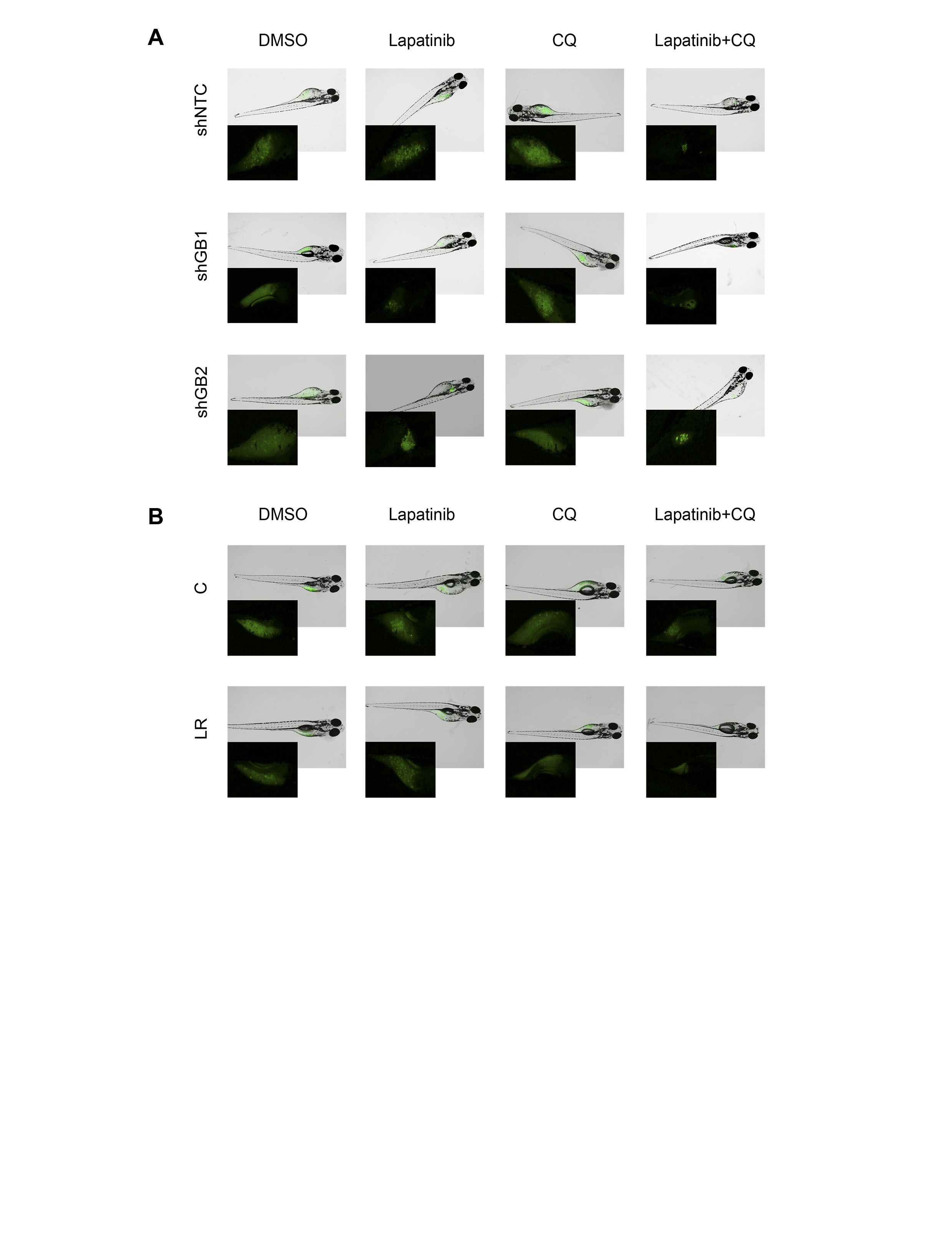
**

**Supplementary Figure 6.** The combination of lapatinib plus chloroquine increases the therapeutic response *in vivo* in zebrafish xenografts of GSDMB-expressing tumors**. A-B:** Representative fluorescence stereomicroscope images of GFP expressing control (shNTC), and GSDMB-silenced (shGB1 and shGB2) HCC1954 xenografts (**A**) or HCC1954 LR and control (C) tumors (**B**) treated with the LC50 of lapatinib and/or chloroquine (35,1 mM and 116,4 mM, respectively). Insets represent an augmented image of GFP-positive tumor. NTC, non-targeting control. LR: Lapatinib resistant cells. CQ, Chloroquine.


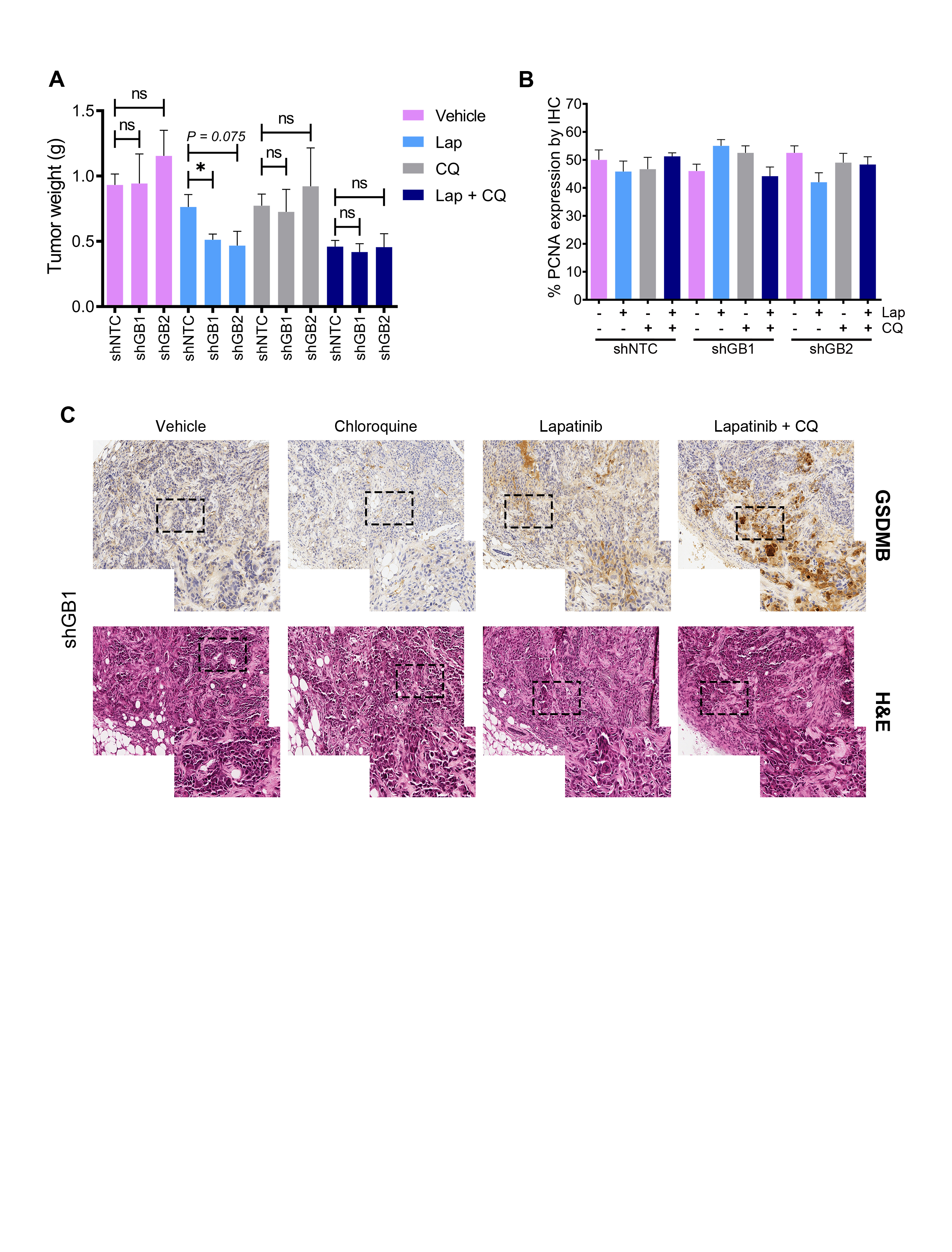


**Supplementary Figure 7.** Immunohistochemical and histological analysis in orthotopic tumor xenografts. **A:** Quantification of tumor weight from the mouse experiment presented in Figure 4D ordered by groups of treatment. **B:** Quantification of PCNA immunohistochemical expression on HCC1954 control (shNTC), or GSDMB-silenced cells (shGB1 and shGB2) xenografted tumors treated with lapatinib, Chloroquine (CQ), or a combination of both. **C:** Representative images of GSDMB immunohistochemical analysis and hematoxylin and eosin staining in shGB1 xenografted tumors, treated with the different therapeutic strategies indicated in (**A**). Immunohistochemical images was taken on 10X and 40X (insets) magnification. NTC, non-targeting control. CQ, Chloroquine. Lap, lapatinib.


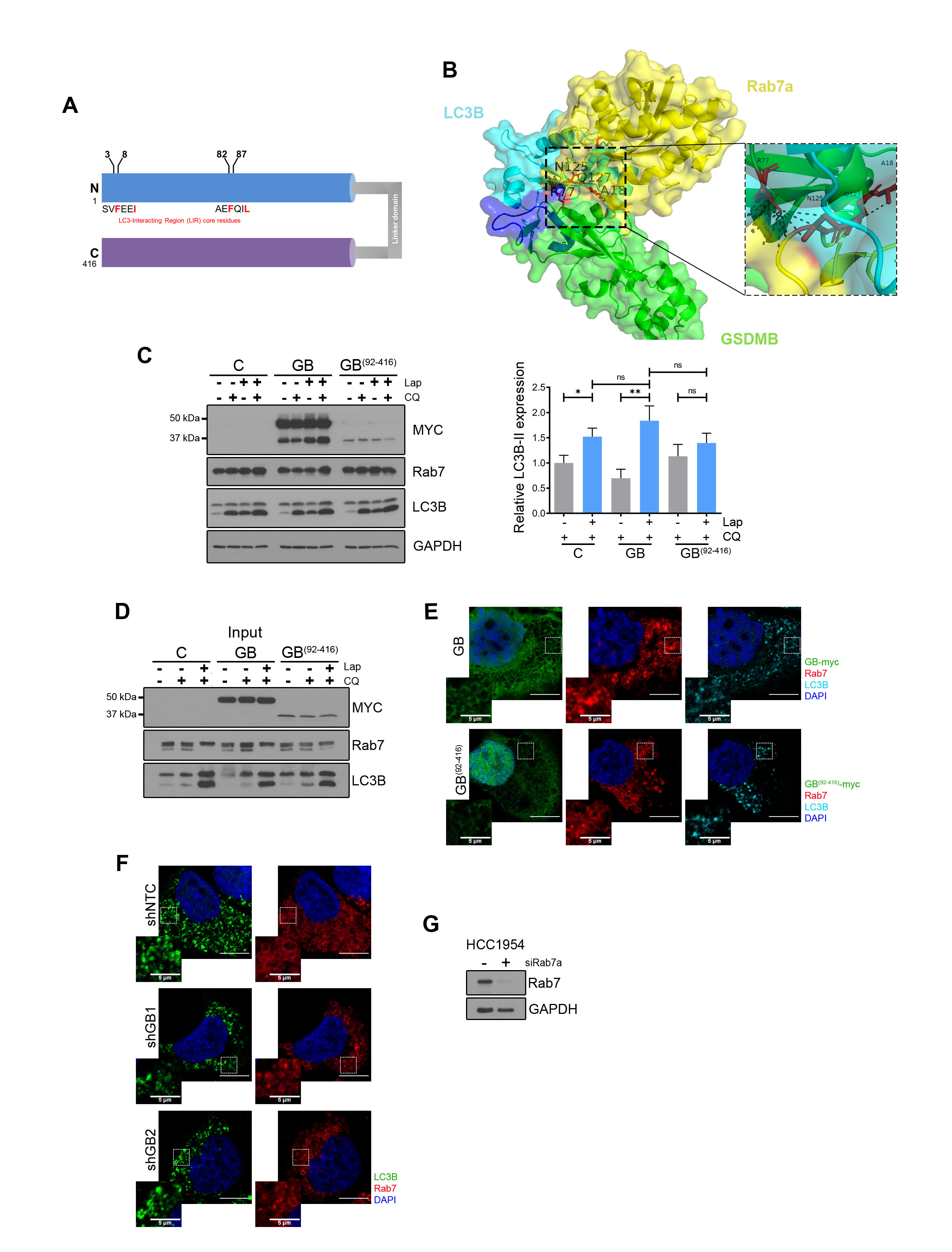


**Supplementary Figure 8.** GSDMB potentially forms a multiprotein complex with Rab7 and LC3B. **A:** Schematic representation of GSDMB showing the LIR motifs (core residues in red) located on the N-terminal region. **B:** *In silico* protein interaction prediction using HawkDock webserver [24] identifies a potential interaction between LC3B (blue), the LIR motif within the GSDMB N-terminal domain (green) and Rab7a (yellow). Inset magnifies the predicting interacting region (red). 3D structures were obtained from Uniprot data bases (Q8TAX9: GSDMB, Q9GZQ8: L3CB and P51149: Rab7a). **C:** Western blot analysis of Myc-tagged GSDMB, Rab7 and LC3B in HCC1954 C, GB and GB^(92-416)^ treated for 72 h with lapatinib (2 µM) and/or CQ (10 µM). Quantification of LC3B-II expression was performed by densitometric scanning and normalized to GAPDH expression. Statistical significance was determined by two-tailed unpaired *t*-test (**P* < 0.05; ***P* < 0.01; ****P* < 0.001; ns, nonsignificant). Data are shown as the mean ± s.e.m. Three independent experiments with similar results were performed. **D:** Western-blot analysis of the input of the co-immunoprecipitation assays represented in the Fig. 5C and D. **E:** Each individual color channel of the images obtained by confocal microscopy represented in the Fig. 5E. **F:** Each individual color channel of the images obtained by confocal microscopy represented in the Fig. 5F. **G:** Rab7 expression was effectively decreased in HCC1954 cells by a specific siRNA (siRab7a), compared to the control. CQ, Chloroquine. Lap, lapatinib.


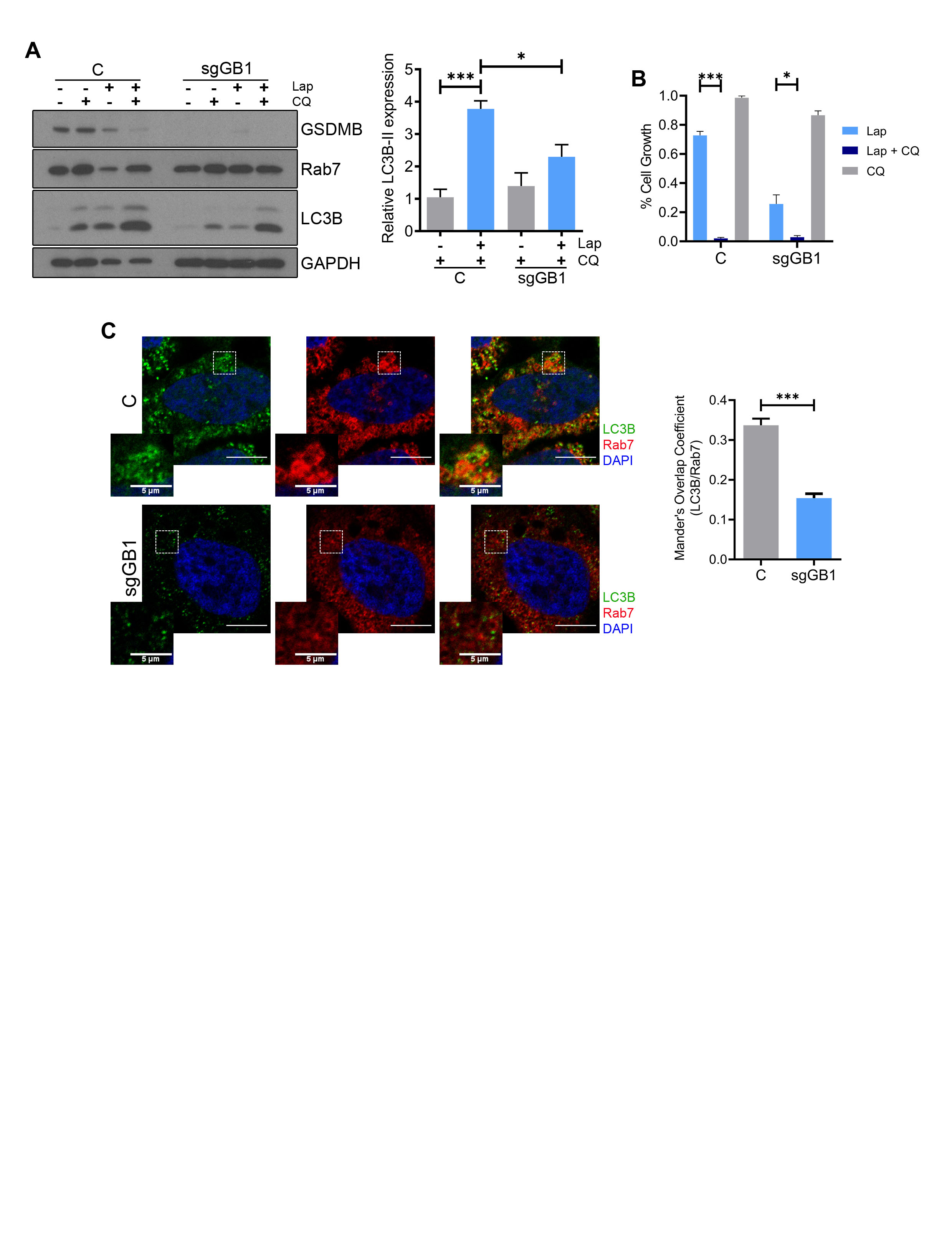


**Supplementary Figure 9.** GSDMB knockout cells show a decreased autophagic flux, correlated with higher sensitivity to lapatinib treatment, compared to control cells. **A:** GSDMB, Rab7 and LC3B protein levels in HCC1954 control (C) and sgGB1 cells treated with lapatinib (2 µM) and/or CQ (10 µM) for 72 h. Quantification of LC3B-II expression was carried out by densitometric scanning and normalized to GAPDH expression. **B:** The cytotoxic effect of the treatment with lapatinib and/or chloroquine in HCC1954 C and sgGB1 cells was evaluated by cell viability assays. **C:** Representative images of the colocalization between Rab7 (red) and LC3B (green) by confocal microscopy in HCC1954 C and sgGB1 cells after lapatinib (2 µM) plus CQ (10 µM) treatment for 72 h. Nuclei were counterstained with DAPI. Quantification of the Manders’ Overlap Coefficient (LC3B overlapping Rab7) is shown on the right. Three independent experiments were performed obtaining at least 60 cells, per experimental condition. Scale bar, 10 µm. Statistical significance was determined by two-tailed unpaired *t*-test (**P* < 0.05; ***P* < 0.01; ****P* < 0.001). Data are shown as the mean ± s.e.m. Three independent experiments with similar results were performed. Lap, lapatinib, CQ, Chloroquine.

**SUPPLEMENTARY TABLES**

**Supplementary Table 1.** Immunohistochemical and clinical data of the HER2+ gastric carcinoma cohort and the HER2+ breast carcinoma series*.

|  | BREAST CANCER*, N (%) | GASTRIC CANCER, N (%) |
| --- | --- | --- |
| GSDMB OVER-expression  Low  High | Reported before* | 13/31 (41.9)  18/31 (58.1) |
| RAB7 puncta expression  Low  High | 22/30 (73.3)  8/30 (26.7) | 9/24 (37.5)  15/24 (62.5) |
| LC3B puncta expression  Low  High | 14/32 (43.8)  18/32 (56.2) | 12/24 (50.0)  12/24 (50.0) |
| Relapse  Low  High | 21/32 (65.6)  11/32 (34.4) | 13/31 (41.9)  18/31 (58.1) |

The dada refers to the available cases for each marker. n (%), number of analyzed cases and (percentage). Relapse, local or distant recurrence. *****GSDMB expression in this breast cancer cohort has been reported before [19].

**Supplementary Table 2**. Relationship between GSDMB expression and clinical or immunohistochemical features in the adjuvant treated HER2-positive gastric carcinoma and breast carcinoma* cohorts.

|  | BREAST CANCER*, N (%) | | | GASTRIC CANCER, N (%) | |
| --- | --- | --- | --- | --- | --- |
|  | **GSDMB**  **LOW** | **GSDMB**  **HIGH** | | **GSDMB**  **LOW** | GSDMB  HIGH |
| RELAPSE  Negative  Positive | Reported  before* | Reported  before* | | 8/13 (61.5)  5/18 (27.8) | 5/13 (38.5)  13/18 (72.2) |
|  |  |  | |  | *p= 0.060* |
| RAB7 puncta expression  Low  High | 15/22 (68.2)  2/8 (25.0) | 7/22 (31.8)  6/8 (75.0) | | 6/9 (66.7)  3/15 (20.0) | 2/9 (33.39)  12/15 (80.0) |
|  |  | *P= 0.045* | |  | *p= 0.011* |
| LC3B puncta expression  Low  High | 12/14 (85.7)  7/18 (35.3) | 2/14 (14.3)  11/18 (64.7) | | 9/12 (75.0)  4/12 (33.3) | 3/12 (25.0)  8/12 (66.7) |
|  |  | | *p= 0.007* | *p= 0.041* | |

The data refer to the available cases for each parameter. ^†^n (%), number of analyzed cases and (percentage). *****GSDMB expression and its association with clinical parameters in this breast cancer cohort has been reported before [19].

**Supplementary Table 3.** *In vivo* acute toxicity results in zebrafish.

|  | **Concentration, µM** | | | |
| --- | --- | --- | --- | --- |
|  | **LC10** | **LC50** | **NOEC*** | **LOEC*** |
| **Lapatinib** | 31.2 | 35.1 | <30.0 | <30.0 |
| **Chloroquine** | 74.1 | 116.4 | 100 | 50 |

*LOEC lowest observed effect concentration, NOEC no-observable effect concentration values

**Supplementary Table 4.** Summary of potential cancer and autophagy GSDMB interactors proteins obtained from immunoprecipitation and mass spectrometry.

**Supplementary Table 5.** Co-expression of GSDMB and autophagy markers (LC3B and Rab7) and their associations with relapse in the adjuvant treated HER2-positive gastric carcinoma and breast carcinoma* cohorts.

|  | BREAST CANCER*, n (%) | | GASTRIC CANCER, n (%) | | |  |
| --- | --- | --- | --- | --- | --- | --- |
|  | **Relapse**  **NO** | **Relapse**  **YES** | | **Relapse**  **NO** | Relapse  YES | |
| GSDMB & RAB7 coexp.  No  YES | 18/24 (75.0)  2/6 (33.3) | 6/24 (25.0)  4/6 (66.7) | | 7/9 (77.8)  6/15 (40.0) | 2/9 (22.2)  9/15 (60.0) | |
|  |  | *P= 0.045* | |  | *P= 0.029* | |
| GSDMB & LC3B COexp.  No  Yes | 11/21 (52.4)  2/11 (18.2) | 10/21 (47.6)  9/11 (81.8) | | 9/10 (90.0)  4/12 (33.3) | 1/10 (10.0)  8/12 (66.7) | |
|  |  | *p= 0.061* | |  | *p= 0.010* | |

The data refer to the available cases for each parameter. ^†^n (%), number of analyzed cases and (percentage). *****GSDMB expression and its association with clinical parameters in this breast cancer cohort has been reported before [19]. Coexp: co-expression.

**Supplementary Table 6.** List of primary antibodies used for western blot (WB), immunofluorescence (IF), immunoprecipitation (IP), and immunohistochemistry (IHC).

| Antibody | Species* | Source | Identifier | WB/IF/IP/IHC |
| --- | --- | --- | --- | --- |
| Anti-ADRP | mMab | Santa Cruz Biotechnology | sc-377429 | -/1:100/-/- |
| anti-ATG5  (C-terminal) | rPab | Sigma-Aldrich | A0731 | 1:500/-/-/- |
| anti-Caspase-3  (Clone 31A1067) | mMab | Santa Cruz Biotechnology | sc-56053 | 1:250/-/-/- |
| anti-GSDMB | mMab | Hergueta-Redondo et al., 2016 | --- | 1:250/-/-/1:200 |
| anti-GAPDH  (Clone 6C5) | mMab | Sigma-Aldrich | CB1001 | 1:50000/-/-/- |
| anti-LC3B | rPab | Sigma-Aldrich | L7543 | 1:1000/1:10/-/1:2000 |
| anti-MAP LC3beta | mMab | Santa Cruz Biotechnology | sc-376404 | -/-/1:100/1:200 |
| anti-Myc-Tag  (Clone 9B11) | mMab | Cell Signaling Technology | 2276 | 1:1000/1:100/-/- |
| anti-Myc-Tag (Clone 71D10) | rMab | Cell Signaling Technology | 2278 | 1:1000/-/-/- |
| anti-HER2  (Clone 44E7) | mMab | Cell Signaling Technology | 2248 | 1:1000/-/-/1:100 |
| anti-Phospo-HER2 (Tyr1221/1222) | rMab | Cell Signaling Technology | 2243 | 1:1000/-/-/- |
| anti-Rab7  (Clone D95F2) | rMab | Cell Signaling Technology | 9367 | 1:500/-/1:100/- |
| anti-Rab7  (Clone E9O7E) | mMab | Cell Signaling Technology | 95746 | 1:1000/1:75/-/1:75 |
| anti-TOM20  (Clone F10) | mMab | Santa Cruz Biotechnology | sc-17764 | -/1:100/-/- |
| anti-SQSTM1/p62 (Clone D5E2) | rMab | Cell Signaling Technology | 8025 | 1:1000/-/-/- |
| PCNA  (Clone p10) | mMab | EMD MILLIPORE | MAB424R | -/-/-/1:10000 |
| HRP-conjugated^#^ | Mouse | GE Healthcare Life Sciences | NA931 | 1:5000/-/-/- |
| HRP-conjugated^#^ | Rabbit | GE Healthcare Life Sciences | NA934 | 1:5000/-/-/- |
| Anti-Mouse IgG (H+L) Alexa Fluor 488-labeled^#^ | Mouse | Invitrogen | A-11029 | -/1:1000/-/- |
| Anti-Mouse IgG2a Alexa Fluor 488-labeled^#^ | Mouse | Invitrogen | A-21131 | -/1:1000/-/- |
| Anti-Mouse IgG1 Alexa Fluor 555-labeled^#^ | Mouse | Invitrogen | A-21127 | -/1:1000/-/- |
| Anti-Rabbit IgG (H+L) Alexa Fluor 546-labeled^#^ | Rabbit | Invitrogen | A-11035 | -/1:1000/-/- |
| Anti-Rabbit IgG (H+L) Alexa Fluor 488-labeled^#^ | Rabbit | Invitrogen | A-11034 | -/1:1000/-/- |

* rPab: rabbit polyclonal antibody, mMab: Mouse monoclonal antibody, rMab: rabbit monoclonal antibody. ^#^ Secondary antibody.

**References**

1. Moreno-Mateos MA, Vejnar CE, Beaudoin JD, Fernandez JP, Mis EK, Khokha MK, et al. CRISPRscan: Designing highly efficient sgRNAs for CRISPR-Cas9 targeting in vivo. Nat Methods. 2015;12:982–8.

2. Molina-Crespo A, Cadete A, Sarrio D, Gamez-Chiachio M, Martinez L, Chao K, et al. Intracellular delivery of an antibody targeting Gasdermin-B reduces HER2 breast cancer aggressiveness. Clin Cancer Res. 2019;25:4846–58.

3. Erbil-Bilir S, Kocaturk NM, Yayli M, Gozuacik D. Study of protein-protein interactions in autophagy research. J Vis Exp. 2017;2017:e55881.

4. Bolte S, Cordelières FP. A guided tour into subcellular colocalization analysis in light microscopy. J Microsc. 2006;224:213–32.

5. Jiang P, Mizushima N. LC3- and p62-based biochemical methods for the analysis of autophagy progression in mammalian cells. Methods. 2015;75:13–8.

6. Yoshii SR, Mizushima N. Monitoring and Measuring Autophagy. Int J Mol Sci. 2017;18:1865.

7. Kovács AL. A simple method to estimate the number of autophagic elements by electron microscopic morphometry in real cellular dimensions. Biomed Res Int. 2014;2014:578698.

8. Vicario R, Peg V, Morancho B, Zacarias-Fluck M, Zhang J, Martínez-Barriocanal Á, et al. Patterns of HER2 Gene Amplification and Response to Anti-HER2 Therapies. PLoS One. 2015;10:e0129876.

9. Westerfield M. The Zebrafish Book. A Guide for the Laboratory Use of Zebrafish (Danio rerio). 5th Edition. University of Oregon Press, Eugene; 2007.

10. Gutiérrez-Lovera C, Martínez-Val J, Cabezas-Sainz P, López R, Rubiolo JA, Sánchez L. *In vivo* toxicity assays in zebrafish embryos: a pre-requisite for xenograft preclinical studies. Toxicol Mech Methods. 2019;29:478–87.

11. Stirling DR, Suleyman O, Gil E, Elks PM, Torraca V, Noursadeghi M, et al. Analysis tools to quantify dissemination of pathology in zebrafish larvae. Sci Rep. 2020;10:3149.

12. Hergueta-Redondo M, Sarrió D, Molina-Crespo Á, Megias D, Mota A, Rojo-Sebastian A, et al. Gasdermin-B promotes invasion and metastasis in breast cancer cells. PLoS One. 2014;9: e90099.

13. Ma Z, Parris AB, Xiao Z, Howard EW, Kosanke SD, Feng X, et al. Short-term early exposure to lapatinib confers lifelong protection from mammary tumor development in MMTV-erbB-2 transgenic mice. J Exp Clin Cancer Res. 2017;36:6.

14. Monma H, Iida Y, Moritani T, Okimoto T, Tanino R, Tajima Y, et al. Chloroquine augments TRAIL-induced apoptosis and induces G2/M phase arrest in human pancreatic cancer cells. PLoS One. 2018;13:e0193990.

15. Jia L, Wang J, Wu T, Wu J, Ling J, Cheng B. In vitro and in vivo antitumor effects of chloroquine on oral squamous cell carcinoma. Mol Med Rep. 2017;16:5779–86.

16. Orellana-Muriana JM. Animal Models in Cancer Research: Assessment of Severity and the Application of Humane Endpoints BT. In Animal Models of Brain Tumors. Neuromethods, vol 77. Humana Press; 2013:21–36.

17. Langford DJ, Bailey AL, Chanda ML, Clarke SE, Drummond TE, Echols S, et al. Coding of facial expressions of pain in the laboratory mouse. Nat Methods. 2010;7:447–9.

18. Ullman-Culleré MH, Foltz CJ. Body condition scoring: a rapid and accurate method for assessing health status in mice. Lab Anim Sci. 1999;49:319–23.

19. Hergueta-Redondo M, Sarrio D, Molina-Crespo Á, Vicario R, Bernadó-Morales C, Martínez L, et al. Gasdermin B expression predicts poor clinical outcome in HER2-positive breast cancer. Oncotarget. 2016;7:56295–308.

20. Jacomin AC, Samavedam S, Promponas V, Nezis IP. iLIR database: A web resource for LIR motif-containing proteins in eukaryotes. Autophagy. 2016;12:1945–53.

21. Waterhouse A, Bertoni M, Bienert S, Studer G, Tauriello G, Gumienny R, et al. SWISS-MODEL: Homology modelling of protein structures and complexes. Nucleic Acids Res. 2018;46:W296–303.

22. Quignot C, Rey J, Yu J, Tufféry P, Guerois R, Andreani J. InterEvDock2: An expanded server for protein docking using evolutionary and biological information from homology models and multimeric inputs. Nucleic Acids Res. 2018;46:W408–16.

23. Sukhwal A, Sowdhamini R. PPcheck: A webserver for the quantitative analysis of protein-protein interfaces and prediction of residue hotspots. Bioinform Biol Insights. 2015;9:141–51.

24. Weng G, Wang E, Wang Z, Liu H, Zhu F, Li D, et al. HawkDock: a web server to predict and analyze the protein-protein complex based on computational docking and MM/GBSA. Nucleic Acids Res. 2019;47:W322–30.

25. Chao KL, Kulakova L, Herzberg O. Gene polymorphism linked to increased asthma and IBD risk alters Gasdermin-B structure, a sulfatide and phosphoinositide binding protein. Proc Natl Acad Sci U S A. 2017;114:E1128–37.
